# Marine-Based Omega-3 Fatty Acids and Metabolic Syndrome: A Systematic Review and Meta-Analysis of Randomized Controlled Trials

**DOI:** 10.3390/nu17203279

**Published:** 2025-10-18

**Authors:** Arghavan Basirat, Juan Francisco Merino-Torres

**Affiliations:** 1 Joint Research Unit on Endocrinology, Nutrition and Clinical Dietetics, Health Research Institute La Fe, 46026 Valencia, Spain; 2 Endocrinology and Nutrition Department, La Fe University Hospital, 46026 Valencia, Spain; 3 Department of Medicine, University of Valencia, 46010 Valencia, Spain

**Keywords:** metabolic syndrome, omega-3 fatty acids, EPA, DHA, triglycerides, fasting blood glucose, insulin resistance, LDL cholesterol, randomized controlled trial, meta-analysis

## Abstract

Background: Metabolic syndrome (MetS) is a set of cardiometabolic abnormalities, including central obesity, dyslipidemia, hypertension, and hyperglycemia, that substantially increases the risk of cardiovascular disease and type 2 diabetes. Marine-derived omega-3 polyunsaturated fatty acids (*n*-3 PUFAs), especially eicosapentaenoic acid (EPA) and docosahexaenoic acid (DHA), may improve MetS components through triglyceride-lowering, anti-inflammatory, and insulin-sensitizing effects; however, randomized controlled trial (RCT) results remain inconsistent, and the influence of dose and intervention duration is unclear. Methods: Following PRISMA guidelines, PubMed, Embase, Scopus, and Web of Science were searched to June 2024 for RCTs in adults with MetS or its components. Eligible trials assessed marine-derived omega-3 supplementation (EPA/DHA) versus placebo or control and reported at least one MetS diagnostic criterion (triglycerides, HDL cholesterol, fasting plasma glucose, blood pressure, or waist circumference) or related parameter (LDL cholesterol, HOMA-IR, or HbA1c). Data were extracted in duplicate and quality assessed using the Cochrane Risk-of-Bias Tool. Trials were categorized by dose—low (<1000 mg/day), medium (1000–2000 mg/day), and high (>2000 mg/day)—and duration: short-term (ST; ≤8 weeks), medium-term (MT; >8–12 weeks), and long-term (LT; >12 weeks). Meta-regression using ordinary least squares estimated dose–duration effects. Publication bias was assessed with funnel plots and Egger’s test for outcomes with ≥3 studies. Results: Twenty-one RCTs (*n* ≈ 1950) were included. For triglycerides, the largest reductions occurred in the high-dose LT (−56.78 mg/dL ± 3.44) and ST (−50.873 mg/dL ± 3.04) groups, and MT duration (−41.536 mg/dL ± 4.12), showing that in high doses of omega-3, the beneficial effect of reducing TGs was more prominent in long-term and short-term treatment other than with medium-term duration of treatment. In comparison, the result for medium-dose with MT duration was (−24.93 mg/dL ± 0.464) and for LT duration was (−31.843 mg/dL ± 0.46), all *p* < 0.001. In LDL cholesterol, an increase in the low-dose ST group (+7.04 mg/dL ± 4, *p* < 0.001) and low-dose LT group (+35.525 mg/dL ± 4.33, *p* < 0.001) was observed. In other subgroups, either there were no data available or the number of studies was limited and could not be considered as statistically significant in meta-analysis due to low power. As for HDL cholesterol, FBS, SBP, DBP, waist circumference, BMI, and HOMA-IR, the data extracted from the included studies were not sufficient to be eligible for the meta-analysis. Conclusions: Marine-derived omega-3 supplementation produces substantial triglyceride reductions, especially at doses >2000 mg/day for ≥8 weeks. HDL cholesterol and blood pressure benefits are not consistent, fasting glycemia is largely unaffected, and LDL cholesterol may increase, especially in low doses. High-dose marine omega-3s can be considered as part of dietary strategies for MetS management, with monitoring for LDL changes. Standardized intervention protocols and long-term RCTs are needed to clarify dose and duration–response relationships.

## 1. Introduction

Metabolic syndrome (MetS) is a cluster of conditions that increase the risk of heart disease, stroke, and type 2 diabetes. The primary components of MetS include elevated blood pressure, high blood sugar, excess body fat around the waist, and low HDL cholesterol or elevated triglyceride levels. The prevalence of MetS is increasing globally, driven by rising obesity rates and sedentary lifestyles [1].

Omega-3 fatty acids, particularly eicosapentaenoic acid (EPA) and docosahexaenoic acid (DHA), have been studied extensively for their potential benefits on these metabolic parameters, including biochemical components, hypertension, and body composition. These fatty acids are primarily found in fish and fish oil supplements and have been shown to have anti-inflammatory, anti-thrombotic, and lipid-lowering properties [2,3]. ALA (alpha-linolenic acid) is another type of omega-3 fatty acid that is found in plant-based sources (e.g., flaxseed oil, etc.), and the effects of this type of omega-3 have also been studied [4], but not as extensively as the marine-based omega-3 fatty acids. The human body can convert ALA into EPA and DHA, but the conversion process is inefficient. Thus, the bioavailability of ALA is far less than that of omega-3 fatty acids derived from marine sources such as EPA and DHA [5].

Recent studies have investigated the effects of omega-3 fatty acids on various components of MetS or the related biochemical parameters, including LDL cholesterol, HOMA-IR (insulin resistance), insulin sensitivity, and HbA1c [6,7,8,9]. For instance, omega-3 fatty acids have been shown to reduce triglyceride levels significantly, which is crucial since high triglycerides are a major risk factor for cardiovascular disease [10]. Additionally, omega-3 supplementation has been associated with improved insulin sensitivity, potentially lowering the risk of developing type 2 diabetes [8,10].

The mechanisms by which omega-3 fatty acids exert these beneficial effects include modulating lipid metabolism, reducing inflammation, and influencing gene expression related to metabolic processes. Despite these promising findings, the effects of omega-3 fatty acids on other MetS components, such as HDL cholesterol and blood glucose levels, have shown mixed results across different studies [11].

To provide a comprehensive understanding of the impact of omega-3 fatty acids on MetS, we conducted a systematic review and meta-analysis focused exclusively on marine-based omega-3 supplementation in adults with MetS or its components. We aimed to quantify the effects of omega-3 on the principal diagnostic criteria for MetS, including triglycerides, HDL-C, fasting blood glucose, and blood pressure, using relevant published randomized controlled trials (RCTs). In addition to these diagnostic markers, other relevant metabolic parameters, such as insulin resistance (HOMA-IR) and LDL-C, were included to provide a broader cardiometabolic profile. We also explored how intervention dose and duration may have affected these biochemical parameters.

In summary, understanding the effects of omega-3 fatty acids on MetS components is essential for developing effective dietary and lifestyle strategies to manage and potentially reduce the risk of MetS [12].

## 2. Method

### 2.1. Objective

This systematic review and meta-analysis follows the Preferred Reporting Items for Systematic Reviews and Meta-Analyses (PRISMA) guidelines.

### 2.2. Search Strategy

A comprehensive search strategy was used to search for relevant studies about omega-3 fatty acids and metabolic syndrome. The databases we searched through were as follows: PubMed (Ovid), Embase, Scopus (Elsevier), and Web of Science (Clarivate). The time span selected included studies published from 2000 up to June 2024. A total of 187 studies were found, of which 21 met the inclusion criteria. The search strategy included keywords like “omega-3 fatty acids,” “metabolic syndrome,” “cardiovascular diseases,” “hypertension,” “insulin resistance,” “type 2 diabetes,” “FBS,” “dyslipidemia,” “obesity,” “waist circumference,” “visceral fat,” “EPA,” “DHA,” “triglycerides,” “cholesterol,” “inflammation,” and “randomized controlled trial.” The search also utilized MeSH terms, including “omega-3 fatty acids” and “insulin resistance,” “omega-3 fatty acids” and “weight,” and “omega-3 fatty acids” and “cardiovascular disease.”

### 2.3. Study Selection

The study selection process followed a multi-stage approach. Two reviewers independently screened titles and abstracts based on the following inclusion criteria:

(1) Randomized controlled trials (RCTs) assessing the impact of omega-3 fatty acids on MetS components in adult populations with diagnosed metabolic syndrome or individuals exhibiting at least one of the components of MetS;
(2) Studies reporting outcomes on at least one of the following: LDL cholesterol, triglycerides, HDL cholesterol, HOMA-IR, fasting plasma glucose, or HbA1c;
(3) Studies published or available in English or Spanish.

**Figure 1:**
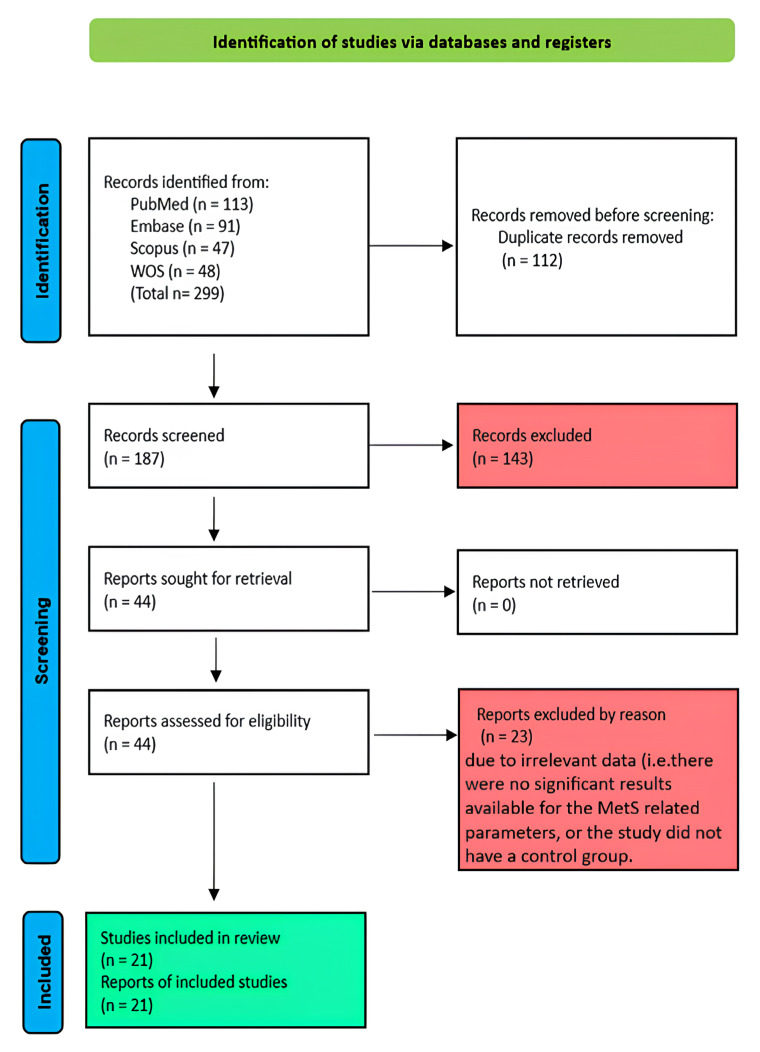
Prisma flowchart.

Initially, duplicates were eliminated, yielding 187 unique articles from a pool of 299 correlated studies. The title and abstract of these articles were screened for relevance, resulting in the exclusion of 143 studies that did not meet the inclusion criteria. The full texts of the remaining 44 studies were then thoroughly reviewed for eligibility according to the inclusion criteria of this study. Of these, 23 studies were excluded for various reasons, such as insufficient relevance to this study’s objectives or lack of adequate data on the outcomes of interest (metabolic Syndrome components or related biochemical parameters). Consequently, 21 studies met the inclusion criteria and progressed to the data-extraction phase. Any discrepancies were resolved through discussion or by involving a third reviewer. The PRISMA flow diagram illustrating this process is shown in Figure 1.

### 2.4. Data Extraction

Data extraction was performed using a standardized form in Excel 2021 by two independent reviewers. The following information was recorded for each study:

Study characteristics: authors, publication year, study design, sample size, and study duration.

Participant characteristics: age, sex, health status, and baseline MetS parameters.

Intervention details: type and dosage of omega-3 fatty acids, comparison group, and duration of intervention.

Outcomes: qualitative summaries and key findings for each MetS component. Discrepancies were resolved through discussion or by consulting the original study.

To enable meta-regression, studies were grouped by the following:

- The omega-3 treatment dose: low dose (LD): <1000 mg/day, medium dose (MD): 1000–2000 mg/day, and high dose (HD): >2000 mg/day;
- The duration of treatment with omega-3 supplementation: short-term (ST): ≤8 weeks, medium-term (MT): >8 to 12 weeks, and long-term (LT): >12 weeks.

### 2.5. Quality Assessment

The quality of the included studies was assessed using the Cochrane Risk-of-Bias Tool version 2. This tool evaluates potential sources of bias, including selection bias, performance bias, detection bias, attrition bias, and reporting bias. Each domain was rated as low, high, or unclear risk of bias. Discrepancies were resolved by consensus.

### 2.6. Data Synthesis and Statistical Analysis

To examine the heterogeneous effects of omega-3 supplementation on various metabolic syndrome parameters, we conducted a secondary quantitative analysis using published results from 21 independent studies.

#### 2.6.1. Study Classification

The studies were first classified into three dosage subgroups according to the administered amount of omega-3: low dose (LD), medium dose (MD), and high dose (HD).

Within each dosage subgroup, studies were further categorized by duration of intervention into short-term (ST), medium-term (MT), and long-term (LT) treatment periods.

#### 2.6.2. Model Specification

For each dosage subgroup and for each metabolic parameter, we estimated the following regression model:

Parameter Change_i_ = β_0_ + β_1_(Treatment)_i_ + β_2_(MT)_i_ + β_3_(LT)_i_ + u_i_

where

- Parameter change represents the observed change in the outcome variable due to omega-3 supplementation.
- Treatment is a dummy variable, coded 1 for the treatment group (omega-3 users) and 0 for the control group.
- MT and LT are dummy variables indicating medium-term and long-term studies, respectively, with short-term groups serving as the baseline category (both MT = 0 and LT = 0).
- u_i_ is the random error term.

Interpretation of the coefficients:

- β_0_ (Intercept) represents the mean baseline change in the studied parameter among the control groups, reflecting the expected variation in the absence of omega-3 supplementation.
- β_1_ (Treatment) measures the mean effect of short-term omega-3 supplementation relative to the control groups. Since Treatment = 1 for treated and 0 for controls, β_1_ indicates the expected change attributable to omega-3 in the short-term (baseline) category, when MT = 0 and LT = 0. A positive and significant β_1_ shows improvement relative to control; a negative and significant β_1_ shows a reduction.
- β_2_ (MT) captures the incremental difference in the parameter change for medium-term interventions compared with short-term ones. A positive and significant β_2_ suggests a stronger omega-3 effect with medium-term use, while a negative and significant β_2_ suggests a weaker effect.
- β_3_ (LT) captures the incremental difference in the parameter change for long-term interventions relative to short-term ones. The sign and magnitude of β_3_ indicate whether prolonged omega-3 use amplifies or attenuates the effect on the metabolic parameter compared with shorter durations.

This model was separately estimated for 12 MetS-related biochemical and metabolic outcomes: LDL, HDL, total cholesterol, fasting blood sugar (FBS), triglycerides, systolic blood pressure (SBP), diastolic blood pressure (DBP), HOMA-IR, insulin, HbA1c, body mass index (BMI), and waist circumference.

#### 2.6.3. Data Simulation and Weighting

Because the original individual-level data of participants were not available, we simulated a weighted dataset from the aggregated results of each paper.

For each study, the reported effect size (mean change due to omega-3) was replicated according to the number of participants in that study.

This procedure assigned greater weight to studies with larger sample sizes and allowed us to approximate individual-level variation when fitting the regression models.

#### 2.6.4. Estimation Procedure

The simulated dataset was analyzed separately within each dosage subgroup (low, medium, and high).

For each subgroup and each metabolic outcome, we estimated the model using ordinary least squares (OLS), including the duration dummy variables (MT and LT).

This approach enabled us to identify the heterogeneous effects of omega-3 across both dosage levels and duration of treatment.

As a robustness criterion, and to minimize the risk of statistical bias, we relied exclusively on the statistically significant effects of omega-3 identified in the OLS regression models. This ensured that the final interpretation of results was based on effects with sufficient empirical support within the analyzed framework.

### 2.7. Assessment of Publication Bias

Publication bias was assessed using funnel plots and Egger’s regression test for outcomes with at least three studies.

However, for outcomes with fewer than three studies, such as blood pressure (systolic and diastolic) and subgroup analyses, funnel plots and Egger’s test were not applied due to methodological limitations of these tools with small sample sizes.

## 3. Results

### 3.1. Study Selection

The PRISMA flow diagram (Figure 1) provides a visual summary of the study selection process. Out of 187 identified records, at the end, 21 studies met the inclusion criteria and were included in the meta-analysis. Studies were excluded for several reasons, including non-RCT design, irrelevant outcomes, unclear source type of omega-3 or plant-based supplementation, and language restrictions.

### 3.2. Study Characteristics

The characteristics of the 21 included studies are summarized in Table 1. The studies varied in sample size, duration, and intervention protocols, providing a broad overview of the different ways omega-3 fatty acids were administered and studied in relation to metabolic syndrome (MetS) components and the related biochemical parameters.

Study Designs: All of the studies are randomized controlled trials (RCTs), which are considered the gold standard in clinical research. They include various approaches, such as double-blind, where neither the participants nor the researchers know who is receiving the treatment, and placebo-controlled designs to ensure unbiased results.

Sample Sizes: There is quite a range in the number of participants across these studies. Some, like Kabir et al. (2007) [9], involved as few as 26 participants, while others, like Gunnarsdottir et al., 2008 [15], had over 250. Most studies fall somewhere in the middle, with sample sizes between 40 and 120 participants.

Age Range: The participants’ ages also vary widely, reflecting the diverse populations these studies seek to understand. Some trials, such as Wong et al. (2013) [13], include younger and middle-aged adults (18–75 years), while others like Ogawa et al. (2013) [10] focus solely on the elderly, targeting patients as old as 90. Many studies, such as DeFina et al. (2011) [2] and Yamamoto et al. (2014) [8], hone in on middle-aged adults with a specific metabolic condition.

Sex Distribution: The sex balance in these studies is also quite varied. While some trials include both men and women in relatively equal numbers, others focus more on one gender. For example, studies like Logan et al. (2015) [21] and Hlavatý et al. (2008) [16] specifically investigated female populations, often targeting post-menopausal women. In studies that include both genders, there is sometimes a notable imbalance, such as the study by DeFina et al. (2011) [2], which had far more female than male participants (40 males and 88 females).

Study Populations: The participants in these trials typically have conditions such as metabolic syndrome, type 2 diabetes, obesity, or dyslipidemia (abnormal cholesterol levels). For example, Yamamoto et al. (2014) [8] focus on older patients with hyperglycemia, while Simão et al. (2014) [25] examine women with metabolic syndrome. Other studies, such as Paoli et al. (2015) [14], investigate the effects of omega-3 when combined with specific diets, such as a ketogenic diet or a weight-loss program.

Interventions: All of these studies look at omega-3 supplementation, sourced from marine (fish oil, krill oil) sources. The interventions vary from simple supplementation, as seen in studies like Kabir et al. (2007) [9] and Mazaherioun et al. (2017) [22], to more complex setups combining omega-3 with other treatments like dietary changes, exercise, or even curcumin (as seen in Thota et al. (2019) [18]). This diversity of interventions allows for a broader understanding of how omega-3 works both alone and in combination with other health-promoting behaviors.

Outcomes: The studies measure a wide range of health outcomes, most commonly focusing on weight loss, insulin sensitivity, blood lipid levels, and other related metabolic parameters. Several studies, such as DeFina et al. (2011) [2], also measure how omega-3 supplementation affects body composition (i.e., muscle vs. fat).

One of the standout features of these studies is how omega-3 is often studied in combination with other lifestyle changes. For instance, DeFina et al. (2011) [2] look at omega-3 along with exercise and a calorie-restricted diet, while Félix-Soriano et al. (2021) [20] investigate its effects in combination with resistance training in post-menopausal women. These combined approaches highlight how omega-3 may boost the effects of other interventions.

### 3.3. Risk of Bias in Studies

**Figure 2:**
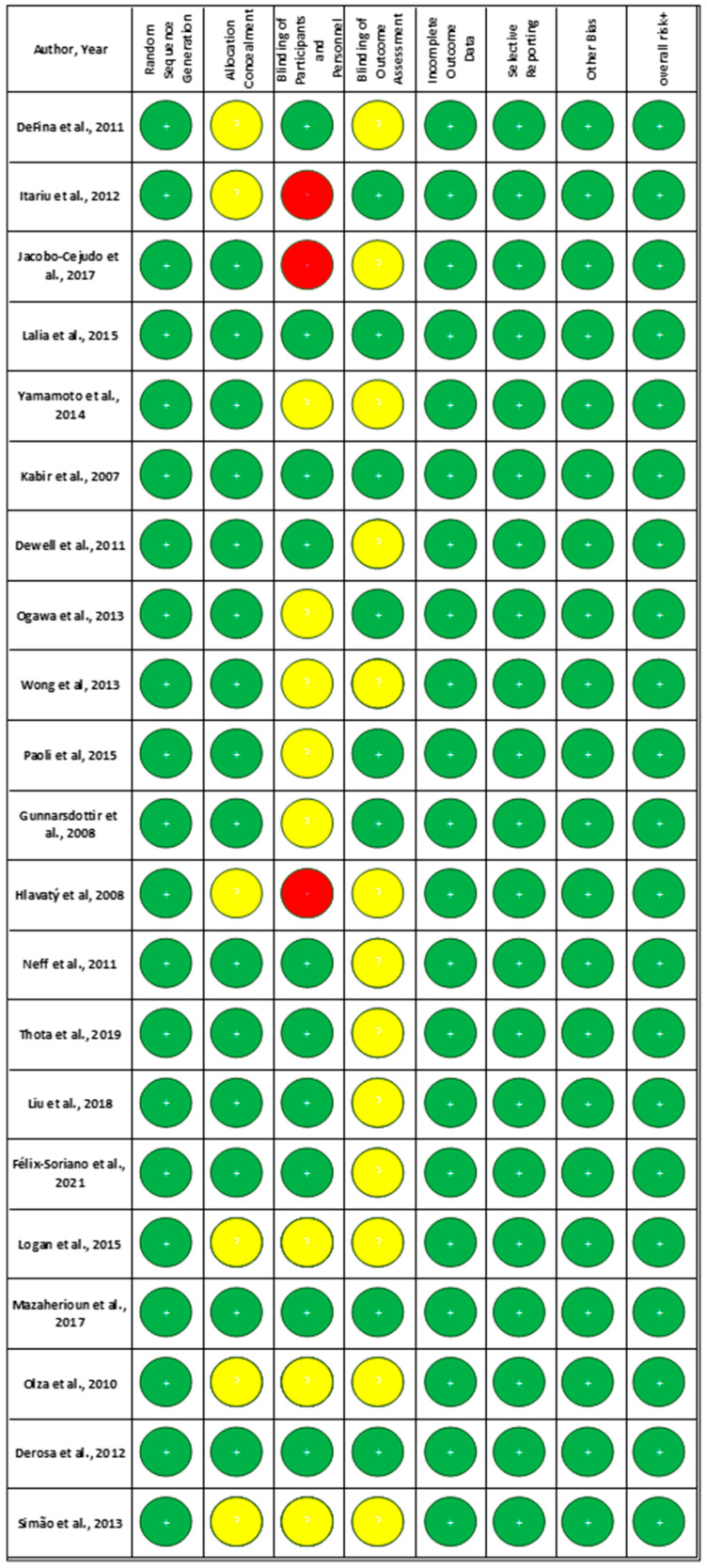
Risk-of-bias assessment for included articles [2,3,6,7,8,9,10,11,13,14,15,16,17,18,19,20,21,22,23,24,25]. Green: Low Risk, Yellow: Unclear risk, Red: High Risk.

**Figure 3:**
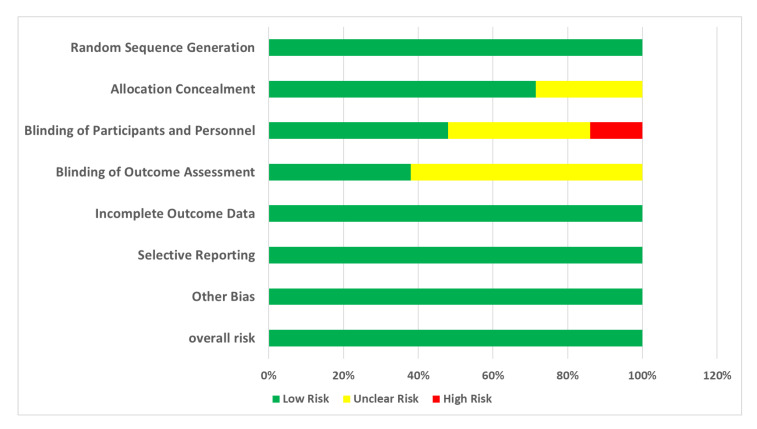
Risk assessment for bias (percentage).

Figure 2 shows the risk-of-bias assessment for each included study. Each trial was evaluated solely using the Cochrane Risk-of-Bias tool. Figure 3 visualizes the proportion of studies rated as having low, unclear, and high risk of bias in each domain.

- **Random Sequence Generation**: All of the studies (100%) were rated as low risk, indicating that proper randomization methods were commonly used;
- **Allocation Concealment**: A significant proportion of studies (71%) were rated as low risk, suggesting that many studies adequately report their allocation concealment methods. However, 29% of studies had an unclear risk due to insufficient information.
- **Blinding of Participants and Personnel**: While a considerable number of studies achieved low risk (48%), a notable portion remained unclear (38%) or high risk (14%), reflecting challenges in maintaining blinding.
- **Blinding of Outcome Assessment**: Similar trends were observed with blinding of outcome assessors, with 38% rated as low risk and 62% as unclear.
- **Incomplete Outcome Data**: All included studies (100%) effectively managed attrition bias.
- **Selective Reporting**: A high percentage of studies (100%) were rated as low risk, indicating comprehensive reporting of outcomes.
- **Other Bias**: In this domain, 100% of studies are rated as low risk due to the elimination of high-risk studies.

### 3.4. Publication Bias in Studies

As mentioned before, the publication bias assessment was conducted by **funnel plots** and **Egger’s regression test** for outcomes with at least three studies, and due to methodological limitations, the test was not applicable to parameters with less than three studies.

No significant asymmetry was observed, and Egger’s test results were non-significant (*p* > 0.05) for key outcomes including triglycerides, HDL-C, FBG, and HOMA-IR.

The Funnel plots and Egger’s regression are shown in Supplementary Figure S1 and Supplementary Table S1.

The chart provides a comprehensive overview of the risk-of-bias assessments conducted for 21 clinical trials included in this systematic review. Each trial was evaluated using the Cochrane Risk-of-Bias tool, which examines seven key domains: (1) **Random Sequence Generation (Selection Bias):** Assesses whether the study used a proper randomization process. (2) **Allocation Concealment (Selection Bias)**: Evaluates if the allocation sequence was adequately concealed to prevent selection bias. (3) **Blinding of Participants and Personnel (Performance Bias)**: Determines if blinding was maintained to prevent performance bias. (4) **Blinding of Outcome Assessment (Detection Bias)**: Assesses whether outcome assessors were blinded to group assignments to avoid detection bias. (5) **Incomplete Outcome Data (Attrition Bias)**: Examines if all data were accounted for, including reasons for dropouts. (6) **Selective Reporting (Reporting Bias)**: Evaluates whether all pre-specified outcomes were reported. (7) **Other Bias**: Identifies any other potential sources of bias.

### 3.5. Results of Individual Studies

Supplementary Table S2 provides an overview of the key findings and qualitative summaries related to each component of metabolic syndrome (MetS) across the included studies.

**Figure 4:**
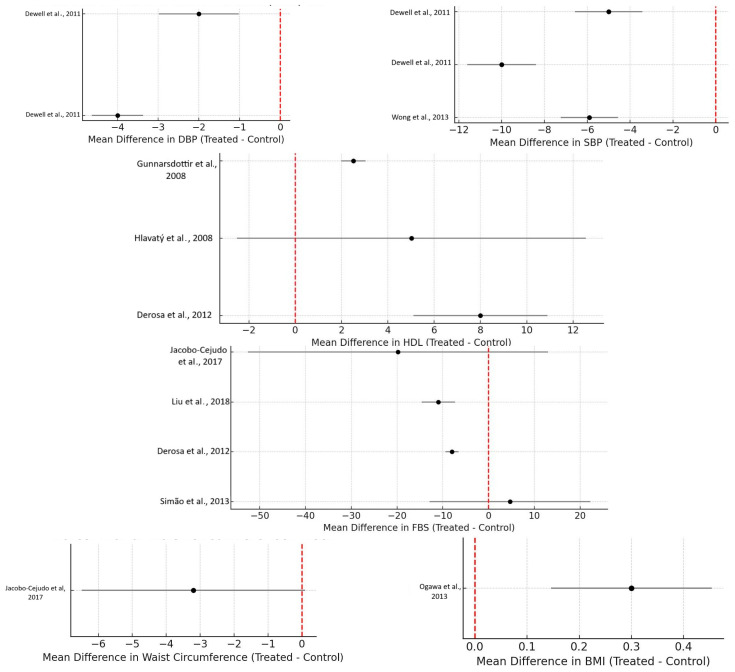

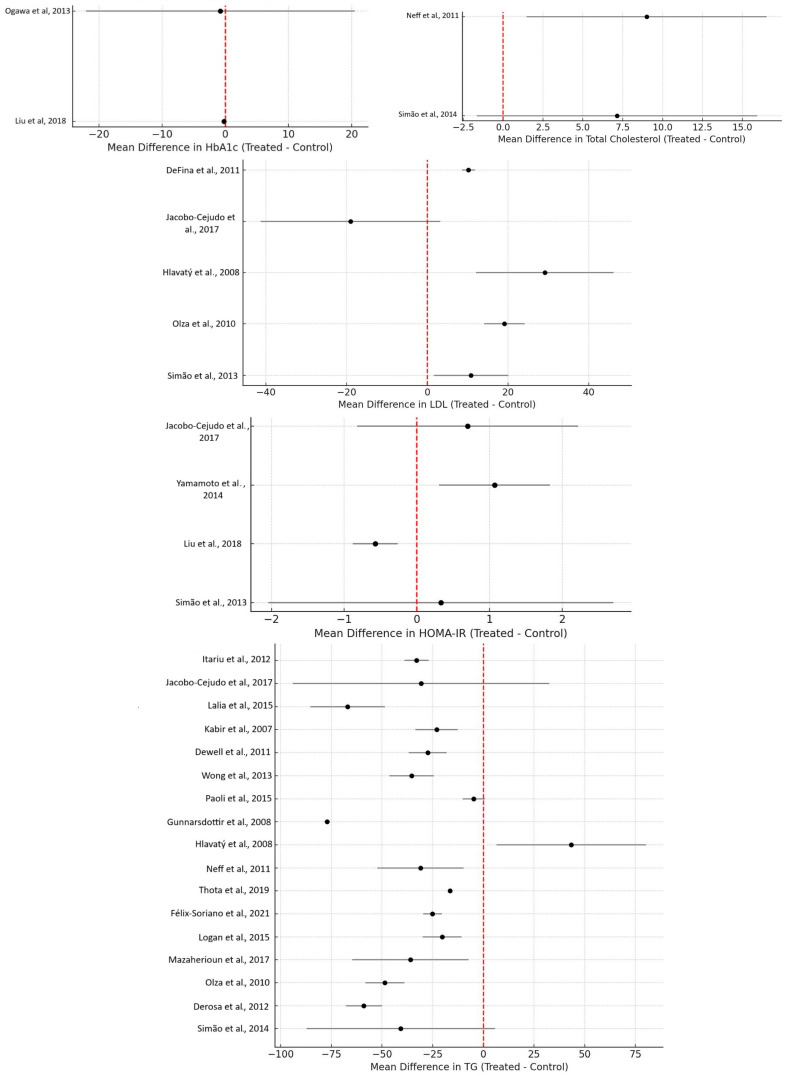
Forest plots of the effect of marine omega-3 supplementation on metabolic syndrome (MetS) parameters and other MetS-related biochemical markers. Each trial is shown with study name, effect size (MD in native units or SMD where required), and 95% CI; square size reflects inverse-variance weight. The red dotted line indicates Zero. Abbreviations: SBP: systolic blood pressure. DBP: diastolic blood pressure. FBS: fasting blood sugar [2,3,6,7,8,9,10,11,13,14,15,16,17,18,19,20,21,22,23,24,25].

Figure 4 provides the Forest plot for the parameters assessed in this systematic review and meta-analysis. This visual representation allows for a better understanding of the direction, magnitude, and statistical significance of the effects of omega-3 supplementation on various metabolic parameters, as well as the consistency of findings across different studies.

- Effects of omega-3 fatty acids on MetS components;
- Triglycerides (TGs).

The impact of omega-3 fatty acids on triglyceride levels was consistently significant across numerous studies:

- Itariu et al. (2012) [3]: a significant reduction in TG levels was observed in non-diabetic, severely obese patients (*p* = 0.03);
- Jacobo-Cejudo et al. (2017) [6]: diabetic patients with a BMI ≤ 30 saw significant reductions in TG levels (*p* = 0.002);
- Lalia et al. (2015) [7]: insulin-resistant, non-diabetic patients exhibited significant reductions in TG levels;
- Kabir et al. (2007) [9]: a significant decrease in triacylglycerol was observed in diabetic, overweight, post-menopausal women (*p* = 0.03);
- Dewell et al. (2011) [11]: fish oil supplementation led to a significant decrease in TG levels compared to flaxseed oil in MetS patients (*p* ≤ 0.01);
- Wong et al. (2013) [13]: obese, dyslipidemic patients in the weight loss + omega-3 group showed a significant reduction in plasma TG compared to the weight loss group alone;
- Paoli et al. (2015) [14]: a significant TG decrease was observed in the KDO3 group compared to the KD group (*p* < 0.05);
- Gunnarsdottir et al. (2008) [15]: overweight and obese young individuals with central obesity experienced a significant reduction in TG levels in all fish and fish oil groups compared to controls (*p* = 0.035);
- Neff et al. (2011) [17]: a significant reduction in total TG concentrations was observed in obese patients with prominent abdominal obesity (*p* = 0.006);
- Thota et al. (2019) [18]: obese individuals with impaired fasting glucose showed a significant reduction in TG levels (*p* < 0.001);
- Félix-Soriano et al. (2021) [20]: a significant reduction in TG levels was observed in overweight and obese post-menopausal women receiving DHA-rich supplementation (*p* = 0.035);
- Logan et al. (2015) [21]: healthy women in the fish oil group experienced a significant 29% reduction in TG levels (*p* = 0.001);
- Mazaherioun et al. (2017) [22]: overweight diabetic patients in the *n*-3 PUFAs group experienced a significant reduction in TG levels from 172 ± 68 mg/dL to 141 ± 54 mg/dL (*p* = 0.039);
- Olza et al. (2010) [23]: patients receiving T-Diet Plus^®^ exhibited a significant decrease in TG levels from baseline (185.5 ± 24.2 mg/dL) to 3 months (132.6 ± 16.7 mg/dL) and 6 months (124.8 ± 15.9 mg/dL) (*p* = 0.002);
- Derosa et al. (2012) [24]: a significant decrease in TG levels was observed in patients with combined dyslipidemia receiving *n*-3 PUFAs (*p* < 0.01);
- Simão et al. (2014) [25]: women with MetS experienced a significant decrease in TG levels in the fish oil group over 90 days (*p* < 0.05).

#### 3.5.1. LDL, HDL, and Total Cholesterol

The effects of omega-3 fatty acids on cholesterol levels were reported as varying across different studies, and omega-3 fatty acids may alter the levels of cholesterol based on the dose and duration of treatment with omega-3. Also, the coexistence of other treatments alongside the omega-3 fatty acids can cause different effects on cholesterol levels.

##### LDL Cholesterol

- DeFina et al. (2011) [2]: an increase in LDL cholesterol levels was noted in obese but otherwise healthy subjects;
- Dewell et al. (2011) [11]: patients with MetS exhibited an increase in LDL levels when treated with fish oil compared to flaxseed oil (*p* ≤ 0.04).

##### HDL Cholesterol

- Hlavatý et al. (2008) [16]: moderately obese non-diabetic women on an omega-3 and low-calorie diet showed a significant increase in HDL levels (*p* < 0.05);
- Neff et al. (2011) [17]: obese patients with prominent abdominal obesity experienced an increase in the concentration of large HDL particles (*p* = 0.001);
- Derosa et al. (2012) [24]: patients with combined dyslipidemia receiving *n*-3 PUFAs showed an increase in HDL levels (*p* < 0.05).

##### Total Cholesterol

- Derosa et al. (2012) [24]: total cholesterol levels decreased in patients receiving *n*-3 PUFAs.
  - **Systolic and Diastolic Blood Pressure**
- In the included studies, the effects of omega-3 on systolic and diastolic blood pressure were mostly insignificant:
- Dewell et al. (2011) [11]: Significant decreases in systolic blood pressure were noted in high-dose fish oil groups compared to the placebo groups in MetS patients (*p* ≤ 0.01). Additionally, diastolic blood pressure decreased significantly compared to all other groups (*p* ≤ 0.02).
- Wong et al. (2013) [13]: a significant reduction in systolic blood pressure was observed in the weight loss + omega-3 group compared to the weight loss group alone (*p* = 0.018).
- Félix-Soriano et al. (2021) [20]: diastolic blood pressure significantly decreased in the DHA-rich supplementation group (*p* = 0.038).
  - **Weight, BMI, and Waist Circumference**

The impact of omega-3 on weight loss, BMI, waist circumference and body fat mass or body lean mass differed vastly depending on the dose and receiving other treatment or lifestyle changes during the study.

- Jacobo-Cejudo et al. (2017) [6]: diabetic patients with a BMI ≤ 30 showed a significant decrease in waist circumference (*p* = 0.001);
- Kabir et al. (2007) [9]: significant decreases in total fat mass were observed in diabetic, overweight, post-menopausal women treated with fish oil.
  - **Fasting Blood Sugar (FBS) and Insulin Sensitivity**
- Among many studies focusing on the effects of omega-3 on fasting blood glucose (FBS), insulin sensitivity, and insulin resistance, there are different contradictory findings depending on the study design:
- Jacobo-Cejudo et al. (2017) [6]: diabetic patients with a BMI ≤ 30 experienced significant reductions in FBS (*p* = 0.011) and HbA1c (*p* = 0.009);
- Ogawa et al. (2013) [10]: a significant decrease in fasting plasma glucose was noted in elderly bedridden patients with type 2 diabetes on enteral nutrition (*p* < 0.01);
- Jacobo-Cejudo et al. (2017) [6]: a significant reduction in insulin levels (*p* < 0.001) and HOMA-IR (*p* < 0.001) was reported in diabetic patients with BMI ≤ 30;
- Lalia et al. (2015) [7]: a modest but significant decrease in hepatic insulin sensitivity was noted in insulin-resistant, non-diabetic patients;
- Yamamoto et al. (2014) [8]: a significant decrease in insulin resistance (HOMA-IR) was reported in a hyperlipidemic population (*p* < 0.05);
- Paoli et al. (2015) [14]: overweight but healthy participants in the keto diet + omega-3 group showed a significant decrease in insulin levels compared to the keto diet group alone (*p* < 0.05);
- Liu et al. (2018) [19]: overweight patients newly diagnosed with diabetes exhibited significant reductions in fasting glucose (*p* = 0.02) and HbA1c (*p* = 0.03) in the omega-3 group;
- Derosa et al. (2012) [24]: insulin resistance (HOMA-IR) significantly decreased in patients with combined dyslipidemia receiving *n*-3 PUFAs (*p* < 0.05).

### 3.6. Meta-Analysis Results

#### 3.6.1. Triglycerides (TGs)

Marine-based omega-3 supplementation significantly reduced triglyceride levels across 17 studies. Meta-analysis showed a moderate and consistent decrease in TGs (SMD: −0.53; 95% CI: −0.69 to −0.37; *p* < 0.001), with low heterogeneity (*I*^2^ = 26%). Subgroup and meta-regression analyses revealed a clear dose- and duration-dependent response.

High-dose (HD: >2000 mg/day) interventions showed a heterogeneous effect depending on treatment duration: short-term (≤8 weeks) treatment with HD omega-3 showed a significant and sharp reduction in TG levels (−50.87 mg/dL; *p* < 0.001), despite the limited duration.

At medium-term (8–12 weeks) treatment, the TG-lowering effect was slightly attenuated (−41.54 mg/dL; *p* < 0.001), suggesting a sustained but not escalating benefit. On the other hand, for the long-term (>12 weeks) high-dose omega-3 treatment, the reduction reached its peak again (−56.78 mg/dL; *p* < 0.001), indicating a potential resurgence or consolidation of efficacy over time. This pattern suggests a nonlinear relationship between duration and response in the high-dose group, with short- and long-term interventions being more effective than medium-term ones.

Medium-dose (MD: 1000–2000 mg/day) interventions showed consistent and significant reductions in medium-term (−24.93 mg/dL (*p* < 0.001)) and long-term treatment with medium-dose omega-3 treatment (−31.84 mg/dL (*p* < 0.001)). None of the included studies reported statistically significant data on TG changes in the short-term, medium-dose treatment.

Low-dose (LD: <1000 mg/day) protocols did not demonstrate significant effects regardless of treatment duration.

Overall, duration stratification confirmed that long-term interventions had the most robust effect, especially in the high-dose group. In contrast, a short-term efficacy was only achieved with high doses, while medium-term responses varied, particularly under high-dose protocols, highlighting the complexity of omega-3′s time-dependent mechanisms of action.

#### 3.6.2. HDL Cholesterol (HDL-C)

The meta-regression of the included studies showed inconclusive effects on HDL cholesterol levels due to limited data across the subgroups.

Subgroup analyses showed the following:

On high-dose (HD: >2000 mg/day) omega-3 fatty acids in short-term (ST) and medium-term (MT) treatments, there were no data available; this means that among the included studies, statistically significant results did not exist. For long-term (LT) treatment with HD omega-3, only one study was available, which reported an increase in HDL-C, but the result could not be generalized due to insufficient data for meta-analysis.

On medium-dose (MD: 1000–2000 mg/day) treatment, no studies were available in any duration subgroup.

On low-dose (LD: <1000 mg/day) treatment, the meta regression on the short-term (ST) subgroup revealed a statistically significant increase in HDL-C (+3.20 mg/dL; *p* < 0.001), but the result was derived from only two studies, which is considered low-power data (LPD); therefore, it was not statistically robust in the meta-regression. In the medium-term (MT) and long-term (LT) subgroups, no studies were available.

Although isolated findings primarily suggested a potential HDL-raising effect of omega-3 in certain subgroups, the overall statistical evidence was insufficient due to a shortage of data, single-study effects, and low statistical power. Therefore, no definitive conclusions can be drawn regarding the impact of omega-3 supplementation on HDL-C, regardless of the treatment duration.

#### 3.6.3. Fasting Blood Glucose (FBG)

In high-dose (HD: >2000 mg/day) treatment over a short-term period, there were no studies available with statistically significant results. In the medium-term subgroup, although a reduction of −11.02 mg/dL was observed, this result was not statistically significant. In long-term (LT) treatment, the subgroup analysis revealed a modest reduction (−6.66 mg/dL); however, this was reported from a single study, which cannot be considered reliable or included in meta-regression.

In medium-dose (MD: 1000–2000 mg/day) treatment, no studies were available in any duration subgroup. In low-dose (LD: <1000 mg/day) omega-3 treatment and in the long-term (LT) subgroup, only one study was available, which could not be included in the meta-regression due to insufficient power. Meanwhile, in the short-term (ST) and medium-term (MT) subgroups, no studies were available.

The overall pooled effect of omega-3 on FBG across 11 studies was non-significant (SMD: −0.10; 95% CI: −0.29 to +0.08; *p* = 0.271), with moderate heterogeneity (*I*^2^ = 52%).

While a few isolated studies reported numerical decreases in FBS, the overall evidence is limited and statistically insignificant.

#### 3.6.4. Blood Pressure

##### Systolic Blood Pressure (SBP)

Marine-based omega-3 supplementation was associated with reductions in systolic blood pressure (SBP) in certain high-dose subgroups; however, the evidence is limited and not statistically significant in meta-regression due to low study count.

Subgroup analyses showed that in high-dose (HD: >2000 mg/day) treatment in the short-term (ST) subgroup, a statistically significant reduction was reported (−8.399 mmHg; *p* < 0.001), but the result was based on only two studies, making it low-power data (LPD) and not eligible for meta-regression. On the other hand, in the medium-term (MT) subgroup, a statistically significant reduction was also observed (−11.820 mmHg; *p* < 0.001), again based on only two available studies (low-power Data). In the long-term (LT) group, no data were available.

In medium-dose (MD: 1000–2000 mg/day) and low-dose (LD: <1000 mg/day) treatment groups in all duration subgroups, either no data were available or only one study, which is not eligible for meta-regression.

Overall, although high-dose omega-3 may reduce SBP in the short- and medium-term, these findings are not conclusive due to limited evidence.

##### Diastolic Blood Pressure (DBP)

For diastolic blood pressure (DBP), the available evidence was insufficient. In all subgroups, across all doses and durations, there were either no studies available or only one study, which is not enough for the meta-regression analysis. Therefore, no conclusion can be drawn about the effects of omega-3 supplementation on DBP.

#### 3.6.5. Waist Circumference and BMI

For BMI, the available evidence was also insufficient. There were no studies available in the high-dose or low-dose groups, and only one study was identified in the medium-dose subgroup. As a result, the data were classified as NED (Not Enough Data), and BMI could not be included in the meta-regression analysis. Therefore, no conclusion can be drawn about the effects of omega-3 supplementation on BMI.

For waist circumference, data were similarly limited. No studies were available in the medium-dose and high-dose groups, while only one study was identified in the low-dose subgroup. This again resulted in an NED classification, making it impossible to include waist circumference in the meta-regression analysis. Accordingly, no conclusion can be made regarding the effect of omega-3 supplementation on waist circumference.

#### 3.6.6. Other MetS-Related Biomarkers

##### Insulin Resistance (HOMA-IR) and HbA1c

At first glance, reviewing the included studies, omega-3 supplementation showed opposing effects on insulin resistance (HOMA-IR) depending on dosage, with improvements at high doses and potential worsening at low doses. However, the strength of these findings is limited by the small number of studies in each subgroup.

Meta-analyses of the effects of omega-3 on insulin resistance in high-dose (HD: >2000 mg/day) treatment in a short-term (ST) subgroup could not be performed because no studies were available. However, in the medium-term (MT) subgroup, a statistically significant reduction in HOMA-IR was observed (−1.337; *p* < 0.001), although this was based on only two studies and is considered low-power data (LPD). In the long-term (LT) subgroup, a smaller reduction (−0.25; *p* < 0.001) was also reported, again from only two studies (indicating LPD).

In the medium-dose (MD: 1000–2000 mg/day) treatment group, no studies with statistically significant data were available in any duration subgroup.

In the low-dose (LD: <1000 mg/day) treatment group, in short-term (ST) and medium-term (MT) subgroups, no studies were available. However, in the long-term (LT) subgroup, a statistically significant increase in HOMA-IR was observed (+0.940; *p* < 0.001), which was based on only two studies again and could not be confirmed in meta-regression due to low power.

For HbA1c, the available data were highly limited across all dosage and duration subgroups. In the high-dose group, no data were available for short-term or long-term interventions, and only one study with significant results was available for the medium-term subgroup, insufficient for meta-regression. In the medium-dose group, no data were available for short- or medium-term durations, and only one study was identified in the long-term subgroup (NED). Finally, no data were available in any of the low-dose subgroups. Consequently, the effect of omega-3 supplementation on HbA1c could not be evaluated.

##### LDL Cholesterol (LDL-C)

Reviewing the included studies showed that omega-3 supplementation was associated with statistically significant increases in LDL cholesterol in specific subgroups, particularly in the low-dose group. Performing meta-regression on the data supported these findings in some of the subgroups.

For subgroup analyses in the high-dose (HD: >2000 mg/day) treatment group in the short-term (ST) and medium-term (MT) subgroups, no studies were available. In the long-term (LT) subgroup, even though a significant increase in LDL-C was observed (+10.284 mg/dL; *p* < 0.001), this result was solely based on two studies, which were not eligible for meta-regression due to low-power data (LPD).

In medium-dose (MD: 1000–2000 mg/day) treatment, no studies were available in any duration subgroups.

In the low-dose (LD: <1000 mg/day), short-term (ST) subgroup, a statistically significant increase in LDL-C was observed (+7.040 mg/dL; *p* < 0.001), and this result was confirmed in meta-regression. In the medium-term (MT) treatment, no studies were available. However, again, in the long-term (LT) treatment with LD omega-3, an even larger increase was observed (+35.525 mg/dL; *p* < 0.001), also statistically significant in the meta-regression.

In summary, low-dose omega-3 supplementation was consistently associated with an increase in LDL-C, particularly in short- and long-term protocols, and these findings were statistically significant even after meta-regression. A similar effect was observed in the high-dose long-term group, although the evidence there remains limited by study count.

## 4. Discussion

This systematic review and meta-analysis assessed the effects of marine-based omega-3 polyunsaturated fatty acid (PUFA) supplementation on key diagnostic and other biochemical components related to metabolic syndrome (MetS) and related cardiovascular biomarkers. Our study uniquely stratified findings by dosage (low, medium, and high) and treatment durations (short-, medium-, and long-term), which enabled a different understanding of the dose–response relationship and time-dependent effects of omega-3 supplementation. Observed short-/medium-/long-term differences may partly reflect lifestyle dynamics; a definitive separation of behavioral vs. pharmacologic effects requires trials that rigorously measure and control lifestyle changes.

### 4.1. Principal Findings

The meta-analysis results confirmed that omega-3 supplementation has a significant triglyceride-lowering effect, particularly at high doses (>2000 mg/day), with the strongest effect observed in long-term interventions. These findings are consistent with multiple previous meta-analyses [12,26,27], which have consistently reported reductions in triglyceride levels following marine omega-3 intake. However, our study adds some new insights by demonstrating heterogeneous effects: high-dose interventions were most effective in short-term and long-term durations, while medium-term effects were somewhat attenuated.

In contrast, the impact on HDL cholesterol was inconclusive. While a minor increase was observed in the low-dose short-term subgroup, this result was based on only two studies and deemed low-powered. Our findings align with the broader literature, which has reported heterogeneous and often insignificant effects of omega-3 on HDL-C levels [26,28].

Fasting blood glucose (FBG) showed no significant changes in any subgroup. This is supported by prior meta-analyses that also found omega-3 intake does not consistently improve glucose control [12,29]. The lack of robust data in medium- and low-dose groups also highlights the need for better-powered studies focusing on glycemic outcomes.

Blood pressure outcomes were notable primarily in the high-dose group. Both short-term and medium-term supplementation led to significant reductions in systolic blood pressure (SBP); however, these effects could not be validated in meta-regression due to low study counts. Our results reflect those of Wang et al. (2023) [12], who reported modest SBP and DBP reductions following omega-3 supplementation [12], though their analysis was not stratified by dosage or duration.

Regarding other MetS-related biomarkers, the findings on insulin resistance (HOMA-IR) showed a contrasting pattern. While high-dose omega-3 reduced insulin resistance, low-dose protocols (especially in long-term subgroups) led to significant increases. Our results align with previous meta-analyses [29]; however, by dividing studies into subgroups based on the dose and duration of treatment, these results support the hypothesis that subtherapeutic dosing may dysregulate glucose-insulin homeostasis—an insight corroborated by Guo et al. (2017), who highlighted inconsistent effects based on circulating *n*-3 PUFA levels [28].

Most notably, LDL cholesterol was significantly increased in both high- and low-dose groups, especially with long-term supplementation. These findings resonate with past evidence that omega-3 can raise LDL-C even while reducing triglycerides [27,28], reinforcing the importance of monitoring lipid profiles during treatment.

### 4.2. Comparison with Previous Research

Prior meta-analyses, such as those by Wang et al. (2023) [12] and Akinkuolie et al. (2011) [29], generally confirm the efficacy of omega-3 in lowering TGs and modestly reducing blood pressure [12,26,27], with inconclusive or null effects on insulin sensitivity and glucose [29]. Our analysis extends these findings by identifying critical thresholds in dosage and duration that mediate these effects. Additionally, the integration of subgroup meta-regression allowed us to assess the robustness and clinical significance of these outcomes.

Unlike prior reviews, our analysis also highlights the potential detrimental effects of low-dose omega-3, particularly on LDL-C and insulin resistance. These findings caution against the assumption that “some omega-3 is better than none,” emphasizing instead the need for therapeutic dosing tailored to clinical targets.

### 4.3. Strengths and Limitations

One of the key strengths of this review is its well-structured methodological approach, which combines dosage–duration stratification with meta-regression to validate the statistical findings. This stratification provides us with the opportunity to delve deeper into the effects of omega-3 fatty acids based on the timing and dosage of treatment, and tailor a customized treatment plan for each patient, taking into account the potential side effects of omega-3 intake.

On the other hand, several limitations should be noted. Some subgroup results were drawn from only one or two studies, which limits their generalizability and prevents their inclusion in meta-regression analyses. In addition, variations in omega-3 formulations (EPA-to-DHA ratios), participants’ baseline metabolic status, and co-interventions such as diet or exercise may have contributed to the observed heterogeneity. Because most trials did not report diet or physical activity in sufficient detail, we could not adjust for them; consequently, lifestyle changes may still confound the results. Finally, none of the included studies assessed LDL particle characteristics (such as size and density), which makes it more difficult to fully interpret the increases seen in LDL-C.

### 4.4. Clinical Implications

Clinically, the findings support the prescription of high-dose omega-3 supplementation (>2000 mg/day) for significant reductions in triglyceride levels and improvements in insulin sensitivity; however, the observed increases in LDL-C with both low- and high-dose protocols necessitate concurrent lipid monitoring. Importantly, low-dose supplementation appears not only ineffective but potentially counterproductive for glycemic control.

Future trials should focus on standardizing dosage forms and comparing the relative efficacy of EPA-only versus EPA + DHA formulations across metabolic and cardiovascular outcomes. Studies should also ensure including sufficient sample sizes, physical activity and lifestyle standardization, and treatment durations in dose–duration meta-regression models.

## 5. Conclusions

This systematic review and meta-analysis confirms that marine-based omega-3 supplementation significantly improves triglyceride levels and insulin sensitivity, particularly at high doses and with long-term use. However, it may also increase LDL cholesterol, especially at low doses or with prolonged intake. The effects on HDL cholesterol, fasting glucose, and blood pressure remain inconsistent or limited by low study power. These findings highlight the importance of appropriate dosing and duration in clinical recommendations and emphasize the need for further high-quality trials to clarify omega-3′s role in metabolic and cardiovascular risk management.

## Figures and Tables

**Table 1:** Summary of the characteristics of the included articles.

Author, Year	Country	Type of Study	Sample Size	Age	Sex Distribution	Extra Information
DeFina et al., 2011 [2]	USA	Placebo-controlled, randomized clinical trial	128 (64 participants in the control group; 64 participants in the treated group)	30–60	40 males and 88 females (20 males and 44 females in each group)	The effects of omega-3 and exercise with a calorie-restricted diet on weight loss and body composition. All the participants received dietary and exercise counseling.
Itariu et al., 2012 [3]	Austria	Randomized, controlled clinical trial	46 (23 participants in the control group; 23 participants in the treated group)	20–65	46 females (23 females in each group)	The study mainly focuses on the effects of omega-3 on adipose tissue and systemic inflammation in obese non-diabetic patients.
Jacobo-Cejudo et al., 2017 [6]	Mexico	Randomized, single-blind, placebo-controlled pilot study	65 (31 participants in the placebo group; 34 participants in the fish oil group)	25–60	15 males and 50 females (5 males and 26 females in the placebo group; 10 males and 24 females in the fish oil group)	The study focuses on the effects of omega-3 on metabolic and inflammatory biomarkers in diabetic patients under treatment with oral medication.
Lalia et al., 2015 [7]	USA	Randomized, double-blind, placebo-controlled study	25 (14 participants in the *n*-3 PUFAs group; 11 participants in the placebo group)	30–40	13 males and 12 females (8 males and 6 females in the *n*-3 PUFAs group; 5 males and 6 females in the placebo group)	Effects of omega-3 fatty acids on insulin resistance in non-diabetic insulin-resistant participants.
Yamamoto et al., 2014 [8]	Japan	Clinical trial Phase II	60 (31 participants in the EPA group; 29 participants in the placebo group)	60–80	31 males and 29 females (18 males and 13 females in the EPA group; 13 males and 16 females in the placebo group)	EPA is one of the *n*-3 PUFAs and, in the study, the effects of EPA on insulin resistance in hyperglycemic patients have been assessed.
Kabir et al., 2007 [9]	France	Randomized, double-blind, parallel, placebo-controlled trial	26 (12 participants in the fish oil group; 14 participants in the placebo group)	40–60	26 females	Assessing whether *n*-3 PUFAs have further impacts on body fat, insulin sensitivity, adipose tissue function (including the production of adipokines and inflammatory and atherogenic factors), and gene expression in type 2 diabetes, as well as other components of metabolic syndrome.
Dewell et al., 2011 [11]	USA	Randomized, double-blind, placebo-controlled trial	100 (20 participants in 5 groups: low-dose flaxseed oil (LFx) group; high-dose flaxseed oil (HFx) group; low-dose fish oil (LFO) group; high-dose fish oil (HFO) group; and placebo group)	40–60	36 females, 64 males (13 males and 7 females in the low-dose flaxseed oil (LFx) group; 12 males and 8 females in the high-dose flaxseed oil (HFx) group; 16 males and 4 females in the low-dose fish oil (LFO) group; 11 males and 9 females in the high-dose fish oil (HFO) group; 12 males and 8 females in the placebo group)	The study compares the effects of plant-based and marine omega-3 on plasma inflammatory markers in adults suffering from metabolic syndrome; in addition, it evaluates the dose-dependent effects of each *n*-3 PUFA on components of MetS and inflammatory markers.
Ogawa et al., 2013 [10]	Japan	Multicenter, prospective, randomized controlled trial	26 (13 participants in the EPA/DHA group; 13 participants in the control group)	70–90	6 males and 20 females (4 males and 9 females in the EPA/DHA group; 2 males and 11 females in the control group)	In the study, the effects of omega-3 were evaluated on the glycemic control of elderly bedridden patients.
Wong et al., 2013 [13]	Australia	Randomized, single-blind intervention trial	25 (12 participants in the weight loss group; 13 participants in the weight loss + omega-3 group)	18–75	14 males, 11 females (the gender distribution in each group was not reported)	The study investigates how n3 fatty acid ethyl ester supplementation affects arterial elasticity in obese adults undergoing a weight loss diet.
Paoli et al., 2015 [14]	Italy	Randomized, controlled, parallel-arm clinical trial	34 (18 participants in the Keto diet group; 16 participants in the Keto diet + omega-3 group)	25–65	34 males	The population of the study was generally healthy (apart from being overweight), and at the end of the study, the effects of omega-3 on cardiovascular risk factors and metabolic factors were assessed.
Gunnarsdottir et al., 2008 [15]	Iceland, Spain, and Ireland	Randomized, controlled intervention trial	262 (61 participants in the control group; 67 participants in the Cod diet group; 72 participants in the Salmon diet group; 62 participants in the fish oil diet group)	20–40	138 males and 186 females (24 males and 36 females in the control group; 29 males and 38 females in the Cod diet group; 36 males and 36 females in the Salmon diet group; 23 males and 39 females in the fish oil group)	The study evaluates the impact of consuming fish (both lean and oily) and fish oil on blood lipid levels during weight loss.
Hlavatý et al., 2008 [16]	Czech Republic	Randomized controlled trial	40 (20 participants in the low-calorie diet group and 20 participants in the low-calorie + omega-3 diet)	40–70	40 females	Effects of low-calorie diet plus omega-3 in non-diabetic overweight women.
Neff et al., 2011 [17]	USA	Randomized, double-blind, placebo-controlled clinical trial	36 (17 participants in the placebo group; 19 participants in the DHA group)	18–65	15 males, 21 females (6 males and 13 females in the DHA group; 9 males and 8 females in the placebo group)	The main goal of the study was to investigate the effects of DHA on lipoprotein particle size distribution in overweight and obese participants.
Thota et al., 2019 [18]	Australia	Randomized, placebo-controlled, double-blind clinical trial	64 (16 participants in the placebo group (PL); 15 participants in the curcumin group (CC); 17 participants in the fish oil group (FO); 16 participants in the curcumin + fish oil group (CC-FO))	30–70	26 males and 38 females (7 males and 9 females in the placebo group; 6 males and 9 females in the curcumin group; 7 males and 10 females in the fish oil group; 6 males and 10 females in the curcumin + fish oil group)	The clinical trial focuses on the effects of omega-3 and/or curcumin on insulin resistance and blood lipids in people with a high risk of type 2 diabetes. We used the data reported in the fish oil arm in this systematic review.
Liu et al., 2018 [19]	China	Randomized, double-blind, parallel-controlled trial	122 (30 participants in the control group; 30 participants in the low-carb, high-protein diet group; 31 participants in the omega-3 group; 31 participants in the low-carb, high-protein diet +omega-3 group)	40–60	61 males and 61 females (15 males and 15 females in the control group; 15 males and 15 females in the low-carb, high-protein diet group; 15 males, 16 females in the omega-3 group; 16 males and 15 females in the low-carb, high-protein diet + omega-3 group)	The effects of a low-carb, high-protein diet with or without omega-3 on metabolic markers and glycemic control were studied in patients newly diagnosed with type 2 diabetes.
Félix-Soriano et al., 2021 [20]	Spain	Randomized double-blind, placebo-controlled trial	71 (20 participants in the placebo (PL) group; 15 participants in the DHA-rich *n*-3 PUFA (*n*-3) group; 20 participants in the placebo + resistance training (PL + RT) group; 16 participants in the DHA-rich *n*-3 PUFA + resistance training (*n*-3 + RT) group)	55–70	71 females	The combination of the resistance training with and without omega-3 was studied. The main goal was to investigate the effects of omega-3 on body composition and cardiometabolic factors in overweight and obese post-menopausal women.
Logan et al., 2015 [21]	Canada	Randomized, single-blind, controlled trial (RCT)	24 (12 participants in the fish oil (FO) group; 12 participants in the placebo (PL) group)	60–76	24 females	Effects of omega-3 on metabolism rate and metabolic markers in older females.
Mazaherioun et al., 2017 [22]	Iran	Randomized, double-blind, placebo-controlled clinical trial	85 (41 participants in the placebo group; 44 participants in the *n*-3 PUFAs group)	30–65	53 males, 32 females (29 males and 15 females in the *n*-3 PUFA group; 24 males and 17 females in the placebo group)	The effects of omega-3 on cardiometabolic and inflammatory markers in diabetic patients were assessed.
Olza et al., 2010 [23]	Spain	Randomized, experimental, prospective, intention-to-treat comparative study	65 (33 participants in the control group; 32 participants in the treatment group)	65 < age	52 females and 13 males (25 females and 7 males in the T-Diet Plus (treatment) group 27 females and 6 males in the Jevity (control) group)	*n*-3 polyunsaturated fatty acids and cardiovascular outcomes in the elderly were studied. All the participants needed enteral nutrition.
Derosa et al., 2012 [24]	Italy	Randomized, double-blind, controlled clinical trial	167 (83 participants in the control group; 84 participants in the *n*-3 PUFAs group)	18–75	82 males and 85 females (41 males and 42 females in the control group; 41 males and 43 females in the *n*-3 PUFA group)	Effects of omega-3 on inflammatory markers and insulin resistance in dyslipidemic patients were studied.
Simão et al., 2014 [25]	Brazil	Randomized, parallel-design, controlled clinical trial	65 (15 participants in the control group; 15 participants in the Kinako group; 19 participants in the fish oil group; 16 participants in the Kinako + fish oil group)	35–60	65 females	The effects of fish oil as a main source of omega-3 with and without Kinako (Soy) on serum lipids and glucose in women with metabolic syndrome were assessed.

Table 1 gives a comprehensive summary of 21 clinical trials that investigate how omega-3 affects various metabolic and cardiovascular health outcomes. These studies were conducted across a range of countries, including the USA, Australia, Japan, Spain, and several European nations like Austria and France. The studies span over two decades, with some dating back to 1999, while others are more recent, published as late as 2024. *n*-3 PUFA: *n*-3 polyunsaturated fatty acids. EPA: eicosapentaenoic acid. DHA: docosahexaenoic acid.

## Data Availability

The data presented in this study are available on request from the corresponding author due to privacy and ethical restrictions.

## Supplementary Materials

The following supporting information can be downloaded at: https://www.mdpi.com/article/10.3390/nu17203279/s1, Figure S1. Funnel Plots: Funnel plots were constructed only for biochemical parameters that demonstrated statistically significant effects in more than two studies. For outcomes supported by fewer studies, specifically those with two studies (Low Power Data, LPD) or a single study (Not Enough Data, NED), funnel plot analysis was not performed, as the limited number of data points precludes a reliable assessment of publication bias; Table S1. Egger’s regression for assessment of Publication Bias: No evidence of Publication Bias was detected (Egger’s test *p*-value < 0.05), In some parameters such as LDL and Total cholesterol in High dose Marine based omega-3, even though the final regression meta-analysis is statistically significant, but due to the limited number of studies can not be considered as statistically significant (Two or less than two studies). In some other studied parameters, there was only one study available and is regarded as NOT ENOUGH data because a meta-analysis can not be executed with only one study available in the scope; Table S2: Key Findings and Qualitative Summaries for each Included study.
