# Supplementary material for: Marine-Based Omega-3 Fatty Acids and Metabolic Syndrome: A Systematic Review and Meta-Analysis of Randomized Controlled Trials
Source: Nutrients. 2025 Oct 18;17(20):3279. doi: 10.3390/nu17203279 (PMC12567179; doi:10.3390/nu17203279)
Supplement: Supplementary file 1 [file nutrients-17-03279-s001.zip › Supplementary Figure S1.pdf]

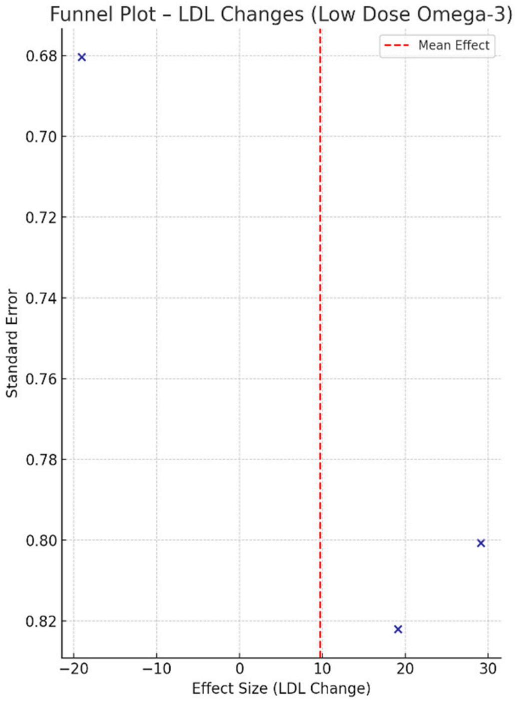
Funnel Plot – LDL Changes (Low Dose Omega-3)
Mean Effect
Standard Error
Effect Size (LDL Change)

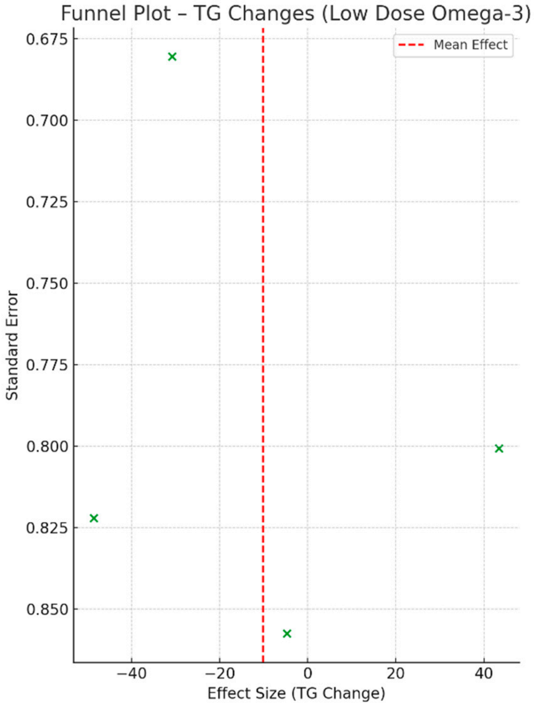
Funnel Plot – TG Changes (Low Dose Omega-3)
Mean Effect
Standard Error
Effect Size (TG Change)

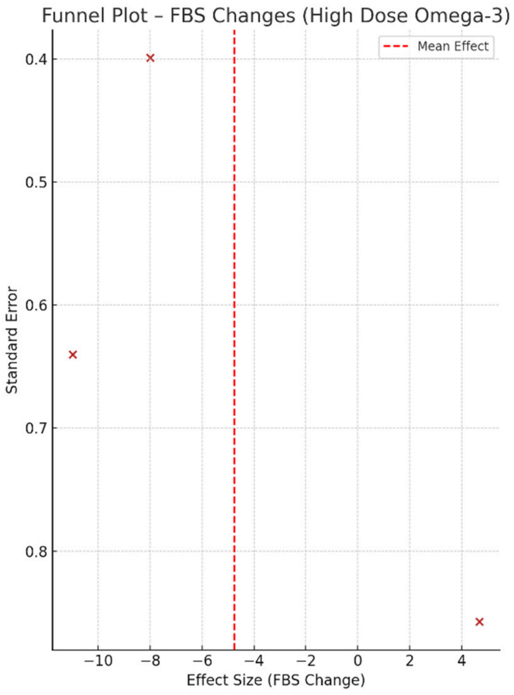
Funnel Plot – FBS Changes (High Dose Omega-3)
Mean Effect
Standard Error
Effect Size (FBS Change)

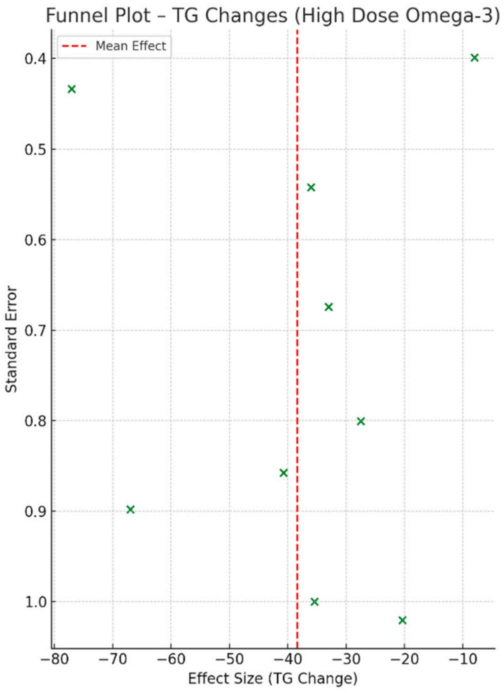
Funnel Plot – TG Changes (High Dose Omega-3)
Mean Effect
Standard Error
Effect Size (TG Change)

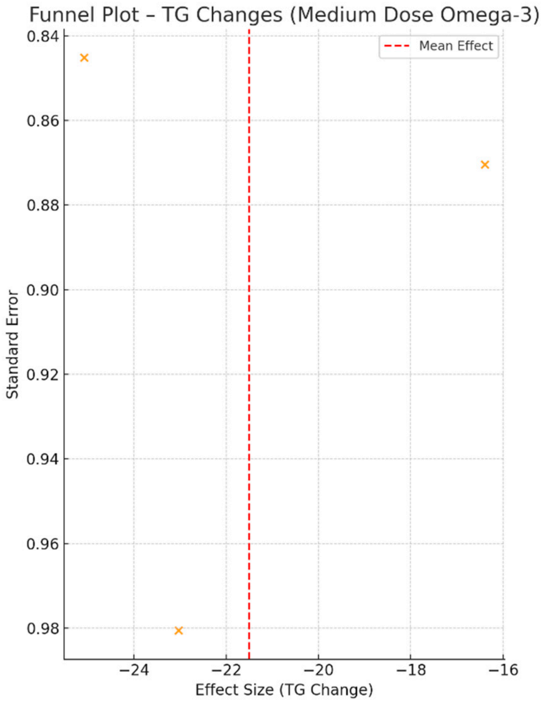

Supplementary Figure S1. Funnel Plots: Funnel plots were constructed only for biochemical parameters that demonstrated statistically significant effects in more than two studies. For outcomes supported by fewer studies, specifically those with two studies (Low Power Data, LPD) or a single study (Not Enough Data, NED), funnel plot analysis was not performed, as the limited number of data points precludes a reliable assessment of publication bias.
