# Supplementary material for: Marine-Based Omega-3 Fatty Acids and Metabolic Syndrome: A Systematic Review and Meta-Analysis of Randomized Controlled Trials
Source: Nutrients. 2025 Oct 18;17(20):3279. doi: 10.3390/nu17203279 (PMC12567179; doi:10.3390/nu17203279)
Supplement: Supplementary file 1 [file nutrients-17-03279-s001.zip › Supplementary Table S2.pdf]

Author, Year	Type and dosage of Omega-3	Duration of the intervention	Health Status of participants	Baseline MetS parameters	Outcomes	Extra Information	final Conclusion
DeFina et al, 2011	**Daily Dose of EPA: 5000 mg**<br>**Daily Dose of DHA: 1000 mg**<br>**Total Daily Omega-3 Fatty Acids: 7000 mg**	168 days (24 weeks)	obese (26 ≤ BMI <40) with no medication or very low-dose medications.	90<Waist circumference<120<br>110<SBP<136 mm Hg<br>67<DBP<88 mm Hg<br>112<LDL<160 mg/DL (25.8% of participants had a history of Hyperlipidemia)<br>80<FBS<100 mg/DL<br>30% of participants had MetS.	LDL cholesterol (mg/dL) increased slightly in the group treated with omega-3. No significant other changes were reported.	Prticipants with conditions or illnesses that could affect study outcomes or make participation potentially harmful, were excluded (metabolic disorders, diabetes, known coronary heart disease, stroke, cancer, hepatic disorders, angina, psychiatric disorders, etc).	This study suggests that lifestyle modification has more impact on weight loss and improvement of MetS parameters in a generally healthy but obese population.
Itariu et al, 2012	**Daily Dose of EPA: 1840 mg**<br>**Daily Dose of DHA: 1520 mg**<br>**Total Daily Omega-3 Fatty Acids: 3360 mg**	56 days (8 weeks)	severely obese (40 ≤ BMI) Fasting plasma glucose<126 mg/dL and 2-hour plasma glucose after a 75-g oral-glucose-tolerance test (OGTT) < 200 mg/dL	40<BMI<52<br>120<SBP<130 mm Hg<br>70<DBP<85 mm Hg<br>115<LDL<137mg/DL<br>90<FBS<100 mg/DL / 5.3%<HbA1c<5.8%<br>The prevalence of MetS is not reported.	Serum triglyceride concentrations (mg/dL) significantly decreased in the n-3 PUFA group. (P = 0.03)	No significant changes were observed in fasting and OGTT-derived serum glucose, insulin, and BMI between the control and n-3 PUFA groups. On the other hand, Plasma IL-6 (pg/mL) concentrations significantly reduced in the n-3 PUFA group. (P=0.04)	Inflammation in adipose tissue underlies the systemic inflammation associated with obesity, increasing the risk of developing metabolic and cardiovascular diseases and this study shows Treatment with long-chain n-3 PUFAs reduces chronic inflammation in subcutaneous and visceral adipose tissues in severely obese, non-diabetic patients.
Jacobo-Cejudo et al, 2017	**Daily Dose of EPA: 320 mg**<br>**Daily Dose of DHA: 200 mg**<br>**Total Daily Omega-3 Fatty Acids: 520 mg**	168 days (24 weeks)	Diabetes and BMI ≤ 30	23<BMI<28<br>SBP N/R<br>SDP N/R<br>126<LDL<166 mg/dL<br>108<BS<245.6 mg/dL<br>6.6<HbA1c<12.7%	Significant decrease in waist circumference (p = 0.001)<br>Significant reduction in glucose (p = 0.011) and HbA1c (p = 0.009)<br>Significant reduction in leptin (p = 0.000) and leptin/adiponectin ratio (p = 0.000)<br>Significant increase in resistin (p = 0.000), insulin (p = 0.000), and HOMA-IR (p = 0.000)<br>Improvement in lipid profile with a significant decrease in triacylglycerides (p = 0.002) and atherogenic index (p = 0.031)	Patients with Hypertension, arthritis, kidney disease, cancer, HIV), fish allergies, insulin treatment, pregnancy, lactation were excluded.	n-3 PUFA supplementation improved waist circumference, glucose, HbA1c, leptin, leptin/adiponectin ratio, and lipid profile in T2DM patients. However, there were increases in resistin, insulin, and HOMA-IR. The study suggests potential benefits of n-3 PUFA supplementation, but higher doses or longer durations may be required for more significant effects. Further research is needed to confirm these findings and explore the combined use of n-3 PUFA and metformin.

Author, Year	Type and dosage of Omega-3	Duration of the intervention	Health Status of participants	Baseline MetS parameters	Outcomes	Extra Information	final Conclusion
Lalia et al, 2015	**Daily Dose of EPA: 2700 mg<br>Daily Dose of DHA: 1200 mg<br>Total Daily Omega-3 Fatty Acids: 3900 mg**	180 days (6 months)	Overweight (BMI > 25 kg/m2) (Insulin-resistant non-diabetic patients)	33<BMI<38<br>SBP N/R<br>SDP N/R<br>91<LDL<116mg/dL<br>88<FBS<96 mg/dL<br>(patients with Diabetes were excluded)	Hepatic insulin sensitivity, determined by the suppression of endogenous glucose production (EGP), showed a modest but significant improvement with EPA+DHA compared to placebo.<br>Triglyceride (mg/dL) decreased significantly compared to the placebo group.	Peripheral insulin sensitivity, measured by hyperinsulinemic-euglycemic clamp, was not significantly changed by EPA+DHA supplementation. Although Hepatic insulin sensitivity, determined by the suppression of endogenous glucose production (EGP), showed a modest but significant improvement with EPA+DHA compared to placebo.	This study suggests that while EPA+DHA may have some beneficial effects on liver insulin sensitivity, they do not significantly impact peripheral insulin sensitivity or overall clinical outcomes related to insulin resistance.
Yamamoto et al, 2014	**Daily Dose of EPA: 900 mg for hyperlipidemic patients 1800 mg for cardiac surgery patients**	180 days (6 months)	Hyperlipidemia (Total cholesterol > 220 mg/dL) patients undergoing cardiac surgery	20<BMI<30<br>SBP N/R<br>SDP N/R<br>90<LDL<155mg/dL<br>85<FBS<155 mg/dL<br>(94% of the patients were dylipidemic, 91.7% of the participants were under treatment for hypertension, and 40% were diabetic)	Significant increase in plasma adiponectin levels (p < 0.05)<br>Significant decrease in insulin resistance (HOMA-IR) (p < 0.05)<br>Significant decrease in Hs-CRP levels (p < 0.05)	In cardiac surgery patients, EPA treatment decreased NLR and antibiotic requirement, suggesting potential anti-inflammatory effects.	EPA administration increased plasma EPA levels and improved insulin resistance and inflammation in hyperlipidemic patients, indicated by increased adiponectin levels and decreased Hs-CRP.
Kabir et al, 2007	**Daily Dose of EPA: 1080 mg<br>Daily Dose of DHA: 720 mg<br>Total Daily Omega-3 Fatty Acids: 1800 mg**	60 days (2 months)	Postmenopausal Women with Diabetes<br>Overweight to obese	28<BMI<32<br>SBP N/R<br>SDP N/R<br>LDL N/R<br>135<FBS<173<br>7.1<HbA1c<8.2	Total fat mass (P = 0.019) and subcutaneous adipocyte diameter (P = 0.0018) were significantly lower in the fish oil group compared to the placebo group.<br>Significant decrease in plasma triacylglycerol (P = 0.03)<br>Significant reduction in the atherogenic index (ratio of triacylglycerol to HDL cholesterol, P = 0.03)	Significant reduction in plasma plasminogen activator inhibitor-1 (PAI-1) (P = 0.01)	A moderate dose of n-3 PUFAs for 2 months reduced adiposity and atherogenic markers without deterioration of insulin sensitivity in women with type 2 diabetes.

Author, Year	Type and dosage of Omega-3	Duration of the intervention	Health Status of participants	Baseline MetS parameters	Outcomes	Extra Information	final Conclusion
Dewell et al, 2011	**Daily dose in Low-dose flaxseed oil (LFx) group: 2.2 grams of alpha-linolenic acid (ALA)**<br>**Daily dose in high-dose flaxseed oil (HFx) group: 6.6 grams of alpha-linolenic acid (ALA)**<br>**Daily dose in Low-dose fish oil group (LFO) :700 mg EPA and 500 mg DHA**<br>**Daily dose in High-dose fish oil group (HFO) :2100 mg EPA and 1500 mg DHA**	56 days (8 weeks)	Patients with MetS (Excluded: BMI ≥ 40, diabetes, renal disease, significant liver enzyme abnormality, pregnancy, lactation, smoking, history of cardiovascular disease, inflammatory disease, malignant neoplasm, clotting disorder, or use of anti-inflammatory, lipid-lowering, or antihypertensive drugs.)	23<BMI<36<br>SBP N/R<br>SDP N/R<br>LDL N/R<br>FBS N/R	LDL Cholesterol: Increased in both fish oil groups compared to both flaxseed oil groups (P ≤ 0.04)<br>Triglycerides: Decreased in the high-dose fish oil group compared to both flaxseed oil groups (P ≤ 0.01)<br>Blood Pressure: Systolic BP decreased in the high-dose fish oil group compared to the high-dose flaxseed and placebo groups (P ≤ 0.01)<br>Diastolic BP decreased in the high-dose fish oil group compared to all other groups (P ≤ 0.02)	The study found no significant reduction in plasma inflammatory markers (MCP-1, IL-6, sICAM-1) with either plant or marine sources of omega-3 fatty acids, regardless of dose, in adults with metabolic syndrome.	there were some beneficial effects on cardiovascular risk factors, such as reductions in triglycerides and blood pressure with high-dose fish oil, although LDL cholesterol increased.
Ogawa et al, 2013	**Approximate Daily Dose of EPA: 670 mg**<br>**Approximate Daily Dose of DHA: 470 mg**	90 days (3 months)	Elderly bedridden patients with type 2 diabetes, and were fed only enteral nutrition through a tube.	16<BMI<24<br>SBP N/R<br>SDP N/R<br>LDL N/R<br>94<FBS<176<br>5.8<HbA1c<8	Significant decrease in fasting plasma glucose (FPG) in EPA/DHA (+) group compared to EPA/DHA (–) group (P < 0.01)<br>Significant decrease in HbA1c in EPA/DHA (+) group compared to EPA/DHA (–) group (P < 0.01)	Significant reduction in fasting remnant-like particles (RLP) in the EPA/DHA (+) group (P < 0.01)<br>Significant decrease in apolipoprotein B (apo-B) in the EPA/DHA (+) group (P < 0.01)<br>Significant reductions in inflammatory markers (TNFα, IL-6, MCP-1) and oxidative stress marker (8-OHdG) in the EPA/DHA (+) group (P < 0.05)<br>No significant changes in total cholesterol, HDL-C, and triglycerides between the two groups	The study concluded that an EPA/DHA-rich diet improved glycemic control and reduced inflammation and oxidative stress in elderly bedridden patients with type 2 diabetes.
Wong et al, 2013	**Daily Dose of EPA: 1840 mg**<br>**Daily Dose of DHA: 1520 mg**<br>**Total Daily Omega-3 Fatty Acids: 3360 mg**	84 days (12 weeks)	Obesity, dyslipidemia (elevated plasma TGs and low HDL cholesterol), and insulin resistance.	26<BMI<40<br>120<SBP<142<br>68<SDP<84<br>88<LDL<159<br>90<FBS<110	Significant reduction in systolic blood pressure in the WL+ omega-3 group in comparison with the WL group (P=0.018)<br>Significant reduction in plasma TG concentration in the WL+ omega-3 group in comparison with the WL group (P=0.032)	Significant reduction in heart rate in the WL+ omega-3 group in comparison with the WL group (P=0.043)<br>Significant increase in stroke volume (P=0.003)	Most of the significant findings in this study were related to Weight loss, but a greater decrease in SBP, and serum TG was noticed in the WL+omega-3 group. according to this study supplementation with omega-3 fatty acid ethyl esters (FAEEs) improves the elasticity of large and small arteries independently of weight loss in obese adults.

Author, Year	Type and dosage of Omega-3	Duration of the intervention	Health Status of participants	Baseline MetS parameters	Outcomes	Extra Information	final Conclusion
Paoli et al, 2015	**Daily Dose of EPA: 115 mg**<br>**Daily Dose of DHA: 65 mg**<br>**Total Daily Omega-3 Fatty Acids: 180 mg**	28 days (4 weeks)	Overweight but otherwise healthy participants.	26<BMI<32<br>SBP N/R<br>DBP N/R<br>117<LDL<150 mg/dL<br>98<FBS<115 mg/dL	Significant triglyceride decrease in the KDO3 group in comparison to the KD group (P< 0.05)<br>Significant Insulin decrease in the KDO3 group in comparison to the KD group (P< 0.05)	Total Cholesterol (TC): Significant decrease in both groups.<br>LDL-C: Significant decrease in both groups.<br>Significant decreases in IL-1β, IL-6, and TNF-α in the KDO3 group	Supplementation with omega-3 fatty acids improved the positive effects of a ketogenic Mediterranean diet on triglycerides, insulin, adiponectin, and inflammatory markers.
Gunnarsdottir et al, 2008	**Cod diet:**<br>**n-3 fatty acids: 272 ± 3 mg/day**<br>**EPA: 54 ± 7 mg/day**<br>**DHA: 207 ± 12 mg/day**<br><br>**Salmon diet group:**<br>**n-3 fatty acids: 3004 ± 129 mg/day**<br>**EPA: 774 ± 29 mg/day**<br>**DHA: 1370 ± 88 mg/day**<br><br>**fish oil diet group:**<br>**n-3 fatty acids: 1418 ± 34 mg/day**<br>**EPA: 633 ± 9 mg/day**<br>**DHA: 430 ± 9 mg/day**	56 days (8 weeks)	Overweight or Obese young adults<br>Waist circumference ≥ 94 cm for men and ≥ 80 cm for women	27.5<BMI<32.5<br>SBP N/R<br>DBP N/R<br>81<LDL<169 mg/dL<br>FBS N/R	Significant reduction in triglycerides (TG) in all fish and fish oil groups compared to control. (P=0.035)	Compliance: Verified through 2-day weighed food records, food frequency questionnaire, and analyzing n-3 and n-6 fatty acids in erythrocyte phospholipids in fasting blood samples.	Weight-loss diet including oily fish resulted in greater TG reduction than a diet without fish or fish oil. The study suggests that including fish in weight-loss diets has beneficial effects on blood lipids, particularly triglycerides, and that the combination of fish proteins and n-3 fatty acids may contribute to these effects.
Hlavatý et al, 2008	**Daily Dose of EPA: 620 mg**<br>**Daily Dose of DHA: 170 mg**<br>**Total Daily Omega-3 Fatty Acids: 790 mg**	21 days (3 weeks)	Moderately obese women<br>Mostly postmenopausal<br>Non diabetic	29<BMI<41<br>SBP N/R<br>DBP N/R<br>116<LDL<198 mg/dL<br>70<FBS<125 mg/dL	Increase in HDL in the n-3 LCD group (p < 0.05)	Higher decrease in LDL-C in the LCD group (p < 0.05)<br><br>Basal Triglyceride levels were significantly higher in the LCD group, and their decrease was higher after weight reduction in the LCD group (p < 0.001)	The study shows that low-dose supplementation of n-3 polyunsaturated fatty acids in yogurt during a short-term weight-reducing regimen increased the proportion of n-3 PUFA in serum lipids and prevented unfavorable changes in serum fatty acid composition. Despite higher basal triglyceride levels in the control group, the n-3 LCD group exhibited beneficial effects on HDL-C and n-3 fatty acid profiles

Author, Year	Type and dosage of Omega-3	Duration of the intervention	Health Status of participants	Baseline MetS parameters	Outcomes	Extra Information	final Conclusion
Neff et al, 2011	**Daily Dose of DHA: 2000 mg**	135 days (4.5 months)	obese and overweight (25≤BMI≤39.9) with prominent abdominal obesity (mean waist circumference 107 cm in men and 99 cm in women).	30<BMI<38<br>89<LDL<146 mg/dL<br>105<SBP<126 mmHg<br>71<DBP<85 mmHg<br>77<FBS<101 mg/dL<br>4.8<HbA1c<5.8%	Reduction in total TG concentrations (p = 0.006)<br>Increase in concentrations of large HDL particles (p = 0.001)	Significant decrease in mean VLDL particle size (p ≤ 0.001)<br>Significant increases in mean LDL (p≤0.001) and HDL (p ≤ 0.001) particle sizes	DHA supplementation resulted in potentially beneficial changes in some markers of cardiometabolic risk, such as VLDL and total triglycerides, and particle sizes of lipoproteins.
Thota et al, 2019	**Total Daily Dose of DHA + EPA: 1200 mg**	84 days (12 weeks)	obese and overweight (25≤BMI≤45) + Impaired Fasting Glucose	29<BMI<33<br>120<LDL<155 mg/dL<br>SBP N/R<br>DBP N/R<br>85<FBS<110 mg/dL<br>30% of the participants were under treatment with antihypertensives.	Triglycerides (TG): Significant reduction in the fish oil (FO) group (P<0.001)	Atherogenic Index of Plasma (AIP):<br>Significant reduction in the curcumin (CC) group (p < 0.05)<br>Significant Insulin Sensitivity (IS) improvement in the curcumin (CC) group (P=0.009) .Insulin Sensitivity (IS)Trends towards improvement in the fish oil (FO) group but not significant.	Fish oil showed significant triglyceride-lowering effects. Combined supplementation of Fish oil and Curcumin did not provide additional benefits over individual supplements.
Liu et al, 2018	**Daily Dose of EPA: 2460 mg<br>Daily Dose of DHA: 690 mg<br>Daily Dose of DPA: 500 mg<br>Daily Dose of ALA: 70 mg<br>Total Daily omega-3 PUFAs: 3650 mg**	84 days (12 weeks)	overweight (18.5≤BMI≤23.9), patients newly diagnosed with Diabetes	18.5≤BMI≤23.9<br>94<SBP<115 mmHg<br>66<DBP<86 mmHg<br>50<LDL<115 mg/dL<br>133<FBS<170 mg/dL<br>6.8<HbA1c<7.5%	Significant reduction in Fasting glucose in the omega-3 group (P=0.02)<br>Significant reduction in HbA1c in the omega-3 group (p=0.03)	Significant reduction in Fasting glucose in the Omega-3+LCHP diet group (P<0.001)<br>Significant reduction in the Omega-3+LCHP diet group (P<0.001)<br>No significant changes in fasting insulin and HOMA-IR across groups.<br>No significant intergroup changes in triglycerides, total cholesterol, LDL-C, and HDL-C.	The combined low-carbohydrate, high-protein diet with omega-3 PUFA supplementation (LCHP+ω-3) provided greater improvements in HbA1c and fasting glucose levels than either diet alone. These findings suggest that combining an LCHP diet with omega-3 supplementation may be an effective strategy for improving glycemic control in newly diagnosed type 2 diabetes patients.

Author, Year	Type and dosage of Omega-3	Duration of the intervention	Health Status of participants	Baseline MetS parameters	Outcomes	Extra Information	final Conclusion
Félix-Soriano et al, 2021	**Daily Dose of EPA: 150 mg**<br>**Daily Dose of DHA: 1650 mg**<br>**Total Daily Omega-3 Fatty Acids: 1950 mg**	112 days (16 weeks)	Overweight and obese postmenopausal women	27.5<BMI<35<br>101<SBP<142 mmHg<br>67<DBP<93 mmHg<br>120<LDL<195 mg/dL<br>85<FBS<130 mg/dL	Significant reduction in diastolic blood pressure in the DHA-rich supplementation group. (p=0.038)<br>Significant reduction in Triglycerides in the DHA-rich supplementation group. (p=0.035)	Fasting glucose and insulin levels showed trends towards improvement, but not statistically significant.<br>DHA-rich supplementation increased muscle quality in lower limbs.<br>Decreases in waist and hip circumferences in all groups.	DHA-rich supplementation had beneficial effects on cardiovascular health markers, including a significant reduction in triglycerides, in overweight/obese postmenopausal women. No synergistic effects were observed for the combination of DHA supplementation and RT program.
Logan et al, 2015	**Daily Dose of EPA: 2000 mg**<br>**Daily Dose of DHA: 1000 mg**	84 days (12 weeks)	Healthy women (Slightly overweight)	25<BMI<30<br>110<SBP<123 mmHg<br>66<DBP<74 mmHg<br>LDL N/R<br>FBS N/R	Significant decrease in triglyceride by 29% in the FO group (P=0.001)	Significant increase in Resting Metabolism Rate by 14% after 12 weeks of fish oil (FO) supplementation.<br><br>Lean Mass: Increased by 4% in the FO group (P=0.01).<br>Heart Rate: Decrease in resting heart rate by 5% (P=0.021).	The study concluded that fish oil supplementation (2 g EPA and 1 g DHA per day) for 12 weeks significantly improved resting and exercise metabolic rates, fat oxidation, lean mass, functional capacity, and reduced triglycerides and heart rate in healthy older females. These findings suggest potential benefits of fish oil supplementation for improving age-related physical and metabolic changes.
Mazaherioun et al, 2017	**Daily Dose of EPA: 1800 mg**<br>**Daily Dose of DHA: 900 mg**<br>**Total Daily Omega-3 Fatty Acids: 2700 mg**	70 days (10 weeks)	Diabetes, Overweight and obese patients	26<BMI<44<br>SBP N/R<br>DBP N/R<br>63<LDL<136<br>129<FBS<236	Triglycerides (TG): Significant reduction in the n-3 PUFAs group from 172 ± 68 mg/dL to 141 ± 54 mg/dL (P=0.039)	Significant reduction in the n-3 PUFAs group (P=0.006)<br>Significant Atherogenic Index improvement in the n-3 PUFAs group (P=0.015)	The study concluded that n-3 PUFA supplementation for 10 weeks significantly reduced serum MCP-1 levels and improved the lipid profile, particularly triglycerides and the atherogenic index, in T2DM patients.

Author, Year	Type and dosage of Omega-3	Duration of the intervention	Health Status of participants	Baseline MetS parameters	Outcomes	Extra Information	final Conclusion
Olza et al, 2010	**Daily Dose of EPA: 94 mg**<br>**Daily Dose of DHA: 44 mg**<br>**Total Daily Omega-3 Fatty Acids: 138 mg**	180 days (6 months)	Requirement for total enteral nutrition (TEN) for at least 6 months	Baseline MetS parameters N/R	Significant decrease in the T-Diet Plus® group from baseline (185.5 ± 24.2 mg/dL) to 3 months (132.6 ± 16.7 mg/dL) and 6 months (124.8 ± 15.9 mg/dL) (P=0.002)	Resistin levels were significantly different between groups (P=0.003).	Feeding elderly patients with an enteral formula enriched with EPA and DHA (T-Diet Plus®) led to significant improvements in their plasma lipid fatty acid profile and lowered triglyceride levels, a known cardiovascular risk biomarker, without affecting insulin resistance markers or adipokine levels. This study suggests that incorporating EPA and DHA into enteral nutrition formulas can be beneficial for improving cardiovascular risk factors in the elderly.
Derosa et al, 2012	**Daily Dose of EPA: 1200 mg**<br>**Daily Dose of DHA: 1350 mg**<br>**Total Daily Omega-3 Fatty Acids: 2550 mg**	180 days (6 months)	combined dyslipidemia (total cholesterol (TC) > 200 mg/dL and triglycerides (TG) > 150 mg/dL)	25<BMI<30<br>120<SBP<140 mmHg<br>78<DBP<90mmHg<br>130<LDL<161mg/dL<br>75<FBS<100 mg/dL	Significant decrease in triglyceride in the n-3 PUFAs group (p<0.01)<br>HDL increased in the n-3 PUFAs group (p<0.05)<br>Insulin Resistance (HOMA-IR) Decreased in the n-3 PUFAs group (p<0.05)<br>Total Cholesterol (TC)decreased in the n-3 PUFAs group	Significant decrease in MMP-2 from 1102.7 ± 132.9 ng/mL to 803.6 ± 78.1 ng/mL (P<0.05 vs baseline).<br>Significant decrease in MMP-9 from 482.9 ± 44.2 ng/mL to 382.7 ± 31.6 ng/mL (P<0.05 vs baseline).	The study concluded that 6 months of n-3 PUFA supplementation at 3 g/day significantly reduced triglycerides, total cholesterol, and several inflammatory markers in dyslipidemicA27:H29 patients. It also improved insulin resistance and decreased levels of metalloproteinases MMP-2 and MMP-9. These findings suggest that n-3 PUFAs can positively affect lipid metabolism and inflammation in dyslipidemic patients.

Simão et al, 2014	**Daily Dose of EPA: 1800 mg<br>Daily Dose of DHA: 1200 mg<br>Total Daily Omega-3 Fatty Acids: 3000 mg**	90 days (3 months)	women with Metabolic Syndrome	25<BMI<43<br>SBP N/R<br>DBP N/R<br>34<LDL<100<br>79<FBS<130	Triglycerides (TG)significantly decreased in the fish oil group from baseline to 90 day (P<0.05)	Significant increase of total cholesterol and LDL in the fish oil group from baseline to 90 days (P<0.05)<br>Significant increase of FBS and insulin sensitivity (HOMA-IR) in the fish oil group from baseline to 90 days (p<0.05)	The study concluded that kinako (29.14 g/day) moderates some adverse effects of high-dose fish oil (3 g/day) on LDL cholesterol, total cholesterol, and glucose metabolism levels. The combination of fish oil and kinako led to improvements in triglycerides and reduced adverse effects on cholesterol and glucose metabolism compared to fish oil alone.

Supplementary Table S2. Key Findings and Qualitative Summaries for each Included study. *presents a detailed summary of clinical trials investigating the effects of omega-3 fatty acids on various metabolic outcomes. The table captures essential aspects such as the type and dosage of omega-3 administered, the duration of interventions, the health status of participants, their baseline metabolic syndrome (MetS) parameters, the outcomes observed, and any extra information or conclusions drawn from each study. This information provides an extensive view of how omega-3 supplementation affects health, particularly for populations with metabolic syndrome, obesity, type 2 diabetes, or cardiovascular risk factors.* ***Omega-3 Types and Dosages:*** *The studies summarized in this table employ varying dosages of omega-3 fatty acids, predominantly focusing on* ***EPA (eicosapentaenoic acid)*** *and* ***DHA (docosahexaenoic acid)****. Dosages range from as low as* ***138 mg/day*** *(e.g.,* ***Olza et al., 2010*** *[22]) to very high doses such as* ***7,000 mg/day*** *in* ***DeFina et al. (2011)*** *[3]. The dosage of omega-3 vary significantly. The diversity in dosage helps to understand omega-3's effects across different concentrations.*

Duration of Intervention: *The trials vary in length, ranging from* ***short-term studies*** *lasting as little as* ***3 weeks*** *(e.g.,* ***Hlavatý et al., 2008*** *[15]) to* ***longer-term interventions*** *extending up to* ***180 days*** *in studies like* ***Olza et al, 2010*** *[22]. The variation in intervention length allows the exploration of both immediate and prolonged effects of omega-3 supplementation.*

Health Status of Participants: *The participants in these studies reflect a wide range of metabolic health conditions. They include individuals with obesity, type 2 diabetes, hypertension, and dyslipidemia. For example:*

- ***DeFina et al. (2011)*** *[3] focuses on* ***obese participants*** *with no or minimal medication, assessing metabolic markers such as blood pressure, LDL cholesterol, and fasting blood glucose.*
- ***Jacobo-Cejudo et al. (2017)****[6] examines individuals with* ***type 2 diabetes*** *(BMI ≤30), highlighting improvements in glucose control, lipid profiles, and insulin sensitivity.*

In some studies, such as ***Ogawa et al. (2013)*** *[11], specialized populations such as elderly bedridden patients with type 2 diabetes were included, while others, like* ***DeFina et al. (2011)*** *[3], focused on overweight but otherwise healthy participants, contributing to a broader understanding of omega-3's effects in different health contexts.*

Baseline Metabolic Syndrome Parameters: *Each study documents baseline metabolic parameters such as BMI, blood pressure (SBP and DBP), cholesterol levels (LDL, HDL, total cholesterol), and fasting blood glucose (FBS), which serve as reference points for evaluating the impact of omega-3 supplementation. For instance,* ***DeFina et al. (2011)***[5] *reports a baseline BMI range of* ***26-40 kg/m²****, with participants having elevated LDL cholesterol (112-160 mg/dL). Other studies, such as* ***Yamamoto et al. (2014)*** *[8], focus on hyperlipidemic patients undergoing cardiac surgery, with baseline total cholesterol levels exceeding* ***220 mg/dL****.*

Outcomes: *The outcomes reported across the studies vary, but common findings include improvements in lipid profiles, insulin sensitivity, and reductions in inflammatory markers. Specific examples include:*

- ***Itariu et al. (2012)*** *[4] showed a significant reduction in* ***serum triglycerides*** *and* ***plasma IL-6 concentrations*** *in severely obese patients after 8 weeks of supplementation.*
- ***Jacobo-Cejudo et al. (2017)*** *[6] demonstrated significant improvements in glucose, HbA1c, and leptin/adiponectin ratios in diabetic patients, while also reporting increases in resistance and insulin levels.*

Using this table, we could divide the included studies into 3 dose-dependent groups and also 3 more groups categorized by the treatment duration.

1. High-Dose Omega-3 Intervention Subgroup:

- ***DeFina et al. (2011)***
- ***Itariu et al, 2012***
- ***Lalia et al, 2015***
- ***Wong et al. (2013)***
- ***Neff et al, 2011***
- ***Liu et al, 2018***
- ***Logan et al, 2015***
- ***Mazaherioun et al. (2017)***
- ***Derosa et al. (2012)***
- ***Simão et al. (2014)***

2. Moderate-Dose Omega-3 Interventions Subgroup:

- ***Kabir et al, 2007***
- ***Ogawa et al, 2013***
- ***Thota et al, 2019***
- ***Félix-Soriano et al, 2021***

3. Low-Dose Omega-3 Interventions Subgroup:

- ***Jacobo-Cejudo et al, 2017***
- ***Yamamoto et al, 2014***
- ***Paoli et al, 2015***
- ***Hlavatý et al, 2008***
- ***Olza et al. (2010)***

Some studies such as Dewell et al, 2011, and Gunnarsdottir et al, 2008 had studied the effects of Omega-3 in both high and low dose treatments.

Conclusions on Effective Doses:

- ***High-Dose Omega-3 (2,000-4,000 mg/day of combined EPA and DHA or more)*** *appears to be the most effective at improving* ***triglycerides***, ***inflammatory markers****, and in some cases,* ***insulin sensitivity****, particularly in high-risk populations (e.g., diabetic or obese patients). Studies like those by* ***Mazaherioun et al. (2017)*** [21] *and* ***Derosa et al. (2012)*** [23] *show clear benefits at these higher dosages.*
- ***Moderate-Dose Omega-3 (around 1,000-2,000 mg/day of combined EPA and DHA)*** *can also provide significant benefits, especially for improving* ***lipid profiles*** *and reducing inflammation in individuals with metabolic syndrome or obesity, as shown in studies like* ***Kabir et al, 2007***
- ***Low-Dose Omega-3 (below 1,000 mg/day)*** *can still yield beneficial outcomes, particularly in specific populations such as elderly patients or those with milder metabolic issues.* ***Olza et al. (2010)*** [22] *demonstrated that even a low dose of omega-3 can lower triglycerides over the long term.*

1. ***Short term Omega-3 Intervention Subgroup:***

- *Itariu et al, 2012*
- *Dewell et al, 2011*
- *Paoli et al, 2015*
- *Gunnarsdottir et al, 2008*
- *Hlavatý et al, 2008*

2. ***Medium term Omega-3 Intervention Subgroup:***

- *Kabir et al, 2007*
- *Wong et al, 2013*
- *Thota et al, 2019*
- *Liu et al, 2018*
- *Logan et al, 2015*
- *Mazaherioun et al, 2017*

3. ***Long term Omega-3 Intervention Subgroup:***

- *DeFina et al, 2011*
- *Jacobo-Cejudo et al, 2017*
- *Lalia et al, 2015*
- *Yamamoto et al, 2014*
- *Ogawa et al, 2013*
- *Neff et al, 2011*
- *Félix-Soriano et al, 2021*
- *Olza et al, 2010*
- *Derosa et al, 2012*
- *Simão et al, 2014*
